# Advancing glioblastoma therapy with surface-modified nanoparticles

**DOI:** 10.1007/s10072-025-08457-4

**Published:** 2025-09-13

**Authors:** Giorgia De Rosa, Marco Zeppieri, Caterina Gagliano, Alessandro Tel, Daniele Tognetto, Pier Paolo Panciani, Marco Maria Fontanella, Tamara Ius, Edoardo Agosti

**Affiliations:** 1 Division of Neurosurgery, Department of Medical and Surgical Specialties, Radiological Sciences and Public Health, University of Brescia, Piazza Spedali Civili 1, Brescia, 25123 Italy; 2 Department of Ophthalmology, University Hospital of Udine, p.le S. Maria della Misericordia 15, Udine, 33100 Italy; 3 Department of Medicine, Surgery and Health Sciences, University of Trieste, Trieste, 34127 Italy; 4 Department of Medicine and Surgery, University of Enna″Kore″, Piazza dell’Università, Enna, 94100 Italy; 5 Eye Center ″G.B. Morgagni-DSV″, Catania, 95125 Italy; 6 Clinic of Maxillofacial Surgery, Head-Neck and NeuroScience Department, University Hospital of Udine, Udine, Italy; 7 Academic Neurosurgery, Department of Neurosciences, University of Padova, Padova, 35121 Italy

**Keywords:** Surface modified nanoparticles, Glioblastoma, Brain-blood barrier, Tumor microenvironment

## Abstract

**Background:**

Glioblastoma multiforme (GBM) is a very aggressive and deadly brain tumor, presenting considerable therapeutic hurdles due to its infiltrative development, heterogeneity, and protective mechanisms of the blood-brain barrier (BBB). Traditional treatment methods frequently do not yield satisfactory results, requiring the implementation of novel solutions. Surface-modified nanoparticles (NPs) have emerged as a viable approach in GBM therapy, providing potential benefits in targeted drug delivery, improved therapeutic efficacy, and reduced systemic toxicity. Aim: This narrative review examines progress in the creation and utilization of surface-modified NPs, emphasizing their function in traversing the blood-brain barrier and selectively targeting glioblastoma cells.

**Methods:**

This review consolidates findings from an extensive search of principal medical databases, highlighting in vitro, in vivo, and ex vivo investigations on surface-modified NPs in the treatment of GBM. The discourse emphasizes diverse methodologies, surface alteration procedures, and their ramifications for therapeutic effectiveness and clinical relevance.

**Results:**

In the last ten years, considerable advancements have been achieved in customizing NPs for targeting GBM. Surface modifications, including conjugation with ligands, peptides, or polymers, have significantly enhanced NP stability, biocompatibility, and specificity. Receptor-mediated targeting has been a primary method, utilizing unique molecular markers that are overexpressed on GBM cells to improve the precision of drug delivery. Dual-targeting strategies that focus on both the blood-brain barrier and tumor microenvironment have demonstrated promise in enhancing therapeutic results. Moreover, sophisticated surface characterization methods have yielded essential insights on NP efficacy, guaranteeing the dependability and consistency of these systems. Preclinical models, especially in vivo studies, have highlighted the translational potential of these methods, showing enhanced medication penetration and efficacy in difficult GBM scenarios.

**Conclusions:**

Surface-modified NPs signify a groundbreaking advancement in GBM therapy, providing novel answers to persistent difficulties. By combining innovative surface engineering with tailored therapeutic administration, they aim to improve treatment accuracy and reduce off-target consequences. Nevertheless, substantial obstacles persist, such as tackling NP toxicity, enhancing surface modification techniques, and guaranteeing scalability for clinical use.

**Supplementary Information:**

The online version contains supplementary material available at 10.1007/s10072-025-08457-4.

## Introduction

Glioblastoma multiforme (GBM) represents 45.6% of primary adult malignant brain tumors, with an annual incidence of 3.1 per 100 000 [1] and a median survival of < 15 months [2–4]. According to the latest World Health Organization (WHO) Classification of Tumors of the Central Nervous System (CNS), published in 2021 [5, 6], all GBMs are now by definition IDH-wildtype, making them the most aggressive form of brain tumors. This, along with difficult drug delivery through the blood-brain barrier (BBB) and with an immunosuppressive tumor microenvironment (TME), explains why current chemotherapies do not yield satisfactory results, requiring novel solutions [7, 8]. New therapeutical strategies, such as immunotherapy, gene therapy, oncolytic virotherapy, stem cell therapy, photodynamic therapy, and hyperthermia therapy [9], are being developed but none of these will have an impact until a way to bypass the BBB and the TME will be found. In this paper, we will explore the challenge of drug transport across the BBB. We will start with a brief overview of this physical and functional barrier to understand how specific receptors on the surface of vascular endothelial cells can be leveraged for their ability to bind ligands, enabling targeted therapy, and facilitating the passage of various substances through the BBB. Among these, surface-modified nanoparticles (NPs) have gained increasing attention over the past decade. Additionally, we will examine the tumor microenvironment (TME) and its intricate crosstalk with GBM stem cells (GSCs), explaining how these emerging drug delivery systems (DDSs) are designed to target tumor cells and disrupt tumor proliferation. Through this narrative review, we aim to provide a comprehensive analysis of the existing literature on these novel DDSs, highlighting their benefits and risks while acknowledging that the lack of a universal marker for effective targeting remains a significant challenge in this field of research.

## Aim

Given the multidisciplinary and rapidly evolving nature of nanoparticle-based therapies for GBM, this review was intentionally focused on surface-functionalized nanoparticles, with particular emphasis on their role in BBB traversal and targeted delivery to the tumor. Several emerging complementary topics—such as theranostic strategies (e.g., LITT, FUS), magnetic hyperthermia, photothermal and photodynamic therapy, radiotherapy-conjugated platforms, patient-derived organoid models, and AI-driven nanoparticle design—are highly relevant to the broader translational landscape, they are beyond the specified scope of this manuscript. We intentionally decided to exclude all these aspects in order to keep the review focused and to provide a deeper discussion of surface modification strategies. However, we recognize the importance of these upcoming novelties and believe they should be addressed in future dedicated reviews.

## Materials and Methods

This narrative review aims to provide a comprehensive synthesis of recent advancements in the use of surface-modified NPs for the treatment of GBM. Given the interdisciplinary nature of this field, the methodology was designed to integrate a broad range of high-quality, relevant sources while maintaining scientific rigor. A systematic Literature search was conducted to identify peer-reviewed articles published up to December 2024. The databases searched included PubMed, Scopus, and Ovid MEDLINE. Search terms were structured around the following keyword combinations: “surface-modified nanoparticles” AND “glioblastoma,” “nanocarriers” AND “blood-brain barrier,” and “tumor microenvironment” AND “targeted drug delivery.” Boolean operators (AND, OR) and Medical Subject Headings (MeSH) were applied as appropriate to refine the search.

Inclusion criteria were as follows: (i) original articles published in English; (ii) studies involving surface-functionalized or surface-modified NPs targeting GBM; and (iii) in vitro or in vivo experimental studies, particularly those addressing BBB penetration, tumor targeting, or modulation of the tumor microenvironment. Exclusion criteria included: (i) non-English publications; (ii) studies that did not explicitly investigate NP surface modifications; and (iii) conference abstracts, editorials, and papers lacking primary data.

Two independent authors screened titles and abstracts to assess relevance to the central topic of NP-based GBM targeting. Full texts of potentially eligible studies were then reviewed in detail. Any disagreements were resolved by consensus. The review focused on research elucidating core principles of NP-cell interactions, ligand-receptor targeting strategies, and their translational potential in GBM models. Findings were thematically summarized across key areas: strategies for physicochemical modification, blood-brain barrier traversal, intratumoral distribution, and therapeutic outcomes.

## Results

The comprehensive literature search conducted for this review Yielded 91 peer-reviewed articles that met the established inclusion criteria. These studies were selected based on their relevance to the role of surface-modified NPs in the treatment of GBM, with particular focus on BBB penetration, tumor specificity, modulation of the TME, and therapeutic efficacy. The publications spanned from 2005 to December 2024, with the majority appearing within the past five years, reflecting growing interest and rapid progress in this interdisciplinary field. Among the selected studies, 37 were exclusively in vitro, 35 utilized in vivo animal models, and 19 employed both experimental approaches. Although most investigations remain at the preclinical stage, they collectively provide substantial insight into the design, performance, and translational promise of surface-engineered NP platforms for GBM therapy.

A central theme across the literature is the critical importance of physicochemical surface engineering in enabling NPs to traverse the BBB. Unmodified NPs are often rapidly cleared and exhibit poor BBB permeability. In contrast, PEGylation has been widely employed to extend systemic circulation and reduce immune recognition. Concurrently, ligand-based functionalization - using agents such as transferrin, lactoferrin, angiopep-2, and folic acid - facilitates receptor-mediated transcytosis across the BBB, enhancing brain accumulation by 2- to 5-fold in preclinical models. Once across the BBB, tumor specificity is achieved through ligands targeting overexpressed receptors such as EGFRvIII, IL13Rα2, and CD44.

Hyaluronic acid-modified NPs exhibit strong affinity for CD44-overexpressing GSCs, thereby reducing therapeutic resistance and recurrence. Similarly, aptamer-mediated targeting of nucleolin and PDGFRβ has demonstrated comparable selectivity. Additional innovations include NPs incorporating MMP-cleavable linkers or pH-responsive coatings that undergo structural transformation within the TME, enhancing intratumoral penetration and distribution. Mannose-functionalized NPs have also shown immunomodulatory potential by reprogramming tumor-associated macrophages (TAMs) from the immunosuppressive M2 phenotype to the pro-inflammatory M1 phenotype, resulting in increased T cell infiltration and tumor suppression. These mechanistically driven surface modifications have led to notable therapeutic outcomes in preclinical evaluations.

Both monotherapy NPs and Dual-delivery systems, such as temozolomide co-delivered with MGMT-targeting siRNA, have consistently outperformed conventional therapies, significantly reducing tumor burden and extending survival in animal models. Median survival in treated cohorts ranged from 38 to 57 days, compared to 22 to 31 days in control groups. Advanced imaging modalities confirmed targeted accumulation within tumors, with tumor-to-brain uptake ratios frequently exceeding 5:1.

Safety profiles across the studies were largely favorable. PEGylated and polysorbate-coated NPs showed no signs of systemic toxicity, and most hematologic and hepatic parameters remained within normal limits. Nonetheless, concerns persist regarding the potential for chronic immunogenicity following repeated administration of ligand-functionalized platforms. Overall, the compiled evidence underscores the pivotal role of surface modification in enhancing the in vivo performance of NPs, governing their pharmacokinetics, biodistribution, tumor targeting, and intracellular delivery.

Despite encouraging preclinical outcomes, the field remains at a critical translational inflection point. Standardization of NP formulations, validation in clinically relevant models, and resolution of regulatory hurdles will be essential for advancing these technologies from the laboratory into clinical practice. The following sections of this review examine these challenges in greater detail.

## Discussion

GBM is a highly heterogeneous tumor, characterized by intricate spatial and temporal heterogeneity that substantially contributes to therapy failure and early recurrence [10, 11]. Despite maximal safe resection and standard chemoradiotherapy, most recurrences arise within 2 cm margin of the first surgery, indicating the persistence of infiltrative tumor cells and the inadequacies of systemic treatments [12]. These challenges have prompted the creation of innovative locoregional treatment techniques designed to improve drug delivery in the peritumoral area and leverage the distinctive biological characteristics of the tumor microenvironment. In this context, surface-modified nanoparticles present a promising approach, providing targeted delivery capabilities and potential integration with existing local therapies to more effectively address residual disease.

### Blood-Brain Barrier and Drug Delivery Mechanisms

Blood-brain barrier is composed of endothelial cells of the capillary wall, astrocyte end-feet, and pericytes [13]. More specifically, it is the result of tight junctions between the endothelial cells of brain capillaries, whose role is to regulate the movement of molecules, ions, and cells between the blood and the CNS [14]. The protective nature of this barrier provides both a defense mechanism from pathogens and toxic substances, and an obstacle for drugs delivery. The structure is further supported by the basal lamina, thick matrix mainly formed by collagen type IV, heparin sulphate proteoglycans, fibronectin, laminin. Multiple basal lamina proteins, matrix metalloproteases (MMPs) and their inhibitors are responsible for the integrity of the BBB and are exploited, due to their dramatically upregulated expression in GBMs, to enhance targeted therapies. Other molecules hyper-expressed in the BBB are transferrin (Tf), insulin, low-density lipoprotein, lactoferrin (Lf), nicotinic receptors, and glucose and choline transporters [15–22]. They have all been studied for GBM treatment, but recent studies proved that targeting a single molecule has limited efficacy. An ideal solution would be to select multiple ligands to increase the chances of crossing the BBB and many studies are focusing, indeed, on multi-ligand functionalized nanomedicine and on dual targeting both the BBB and the tumor cells [23].

Although acting as a selective barrier, the BBB does allow substances to enter, both through gradient-driven or energy-dependent transport. The former represents a passive transport, and it is exactly what current therapeutics take advantage of. However, most of the drugs efficient against GBM do not possess the characteristics to exploit this easy route to the brain, hence the need to exploit different mechanisms, such as para-cellular transport, carrier-mediated transport, or receptor-mediated transcytosis.

The BBB is exploited not only passively, as a semi-permeable barrier creating a so-called “pharmacological sanctuary”, but also actively, through tumor-mediated mechanisms. Increased metabolic demand induces local hypoxia, which leads to overproduction of hypoxia-inducible-factor (HIF). In turn, HIF stimulates vascular endothelial growth factor (VEGF). which alters the BBB architecture and induces the formation of abnormal capillaries. This tumor-regulated neoangiogenesis concurs to accommodate the high metabolic demands of glioma cells, guaranteeing their survival and constant proliferation [24, 25].

### Tumor Microenvironment and Glioblastoma Stem Cells

TME is the region surrounding the primary lesion. It acts as a dynamic and active component of the tumor itself, due to its immunosuppressive nature and its ability to favor metastatic diffusion. The simple existence of these two characteristics led to think that there must be a strong connection between the tumor and its surrounding, connection that was found to lie in the crosstalk between the TME and GSCs. The latter produce neutrosphere-like cell clusters with high CD133 expression that function as trophic agents to the TME, with self-renewal and tumorigenic capacity [26–29]. Among all the GSCs markers, CD133 is one of the most studied due to its association with low survival rates since it can influence recurrence, prognosis and aggressiveness [30–32]. However, GBMs CD133 negative have been identified, proving the heterogeneity that distinguishes this pathology and confirming the lack of a single marker towards which all efforts can be directed. Integrin-α6 and CD44 are other markers often co-expressed with CD133, so most of the studies revolve around them.

Alternatively, another solution is to target the TME instead of GSCs receptors, either by targeting endothelial cells or pathways involved with angiogenesis i.e. VEGFR [33, 34]. Targeting the tumor by exploiting its TME characteristics was a central theme in the 90 s when the enhanced permeation and retention effect (EPR) was first observed. It was basically because of the EPR effect that nanomedicine started to develop to guarantee accumulation at tumor sites [35]. However, recent analysis proved that the EPR effect is more present in rodents than in humans and that it has a considerable heterogeneity among different patients and among different tumors, meaning that it alone cannot guarantee the accumulation of NPs in tumoral cells. Because of this conclusion, NPs have been further modified [36–39]. For example, the addition of collagenase or hyaluronic acid can respectively promote extravasation, following interaction with the CD44 receptor, and improve penetration and accumulation at the tumor site [40].

The epidermal growth factor (EGF) and VEGF, whose role has already been discussed in this paper, represent a target of research because of their hyper-expression in the tumor site. However, the main problem with VEGF, is that anti-angiogenic treatments are often useless in highly innervated tumors. VEGF-targeted NPs could stimulate a response from tumor-associated nerves (TANs) that would annihilate the therapy by restoring angiogenesis [41–43].

Moreover, one of the most predominant cell populations in TME is the one of tumor associated macrophages (TAMs) that have a role in neovascularization, hence in tumor proliferation. These cells are recruited by GSC-induced periostin secretion, meaning that silencing periostin can be considered another valid alternative to target the TME and alter the tumor proliferation.

### Surface-Modified Nanoparticles: Design and Targeting Strategies

NPs are nano-sized molecules whose small sizes, low toxicity and controlled drug release profile [44] won them a key role in recent studies about target therapies against GBM. Additionally, their surface can be modified with targeting ligands, allowing them to localize drug delivery through the blood-brain barrier and into gliomas. Their application in the medical field, known as nanomedicine, allow them to carry traditional drugs to enable passage through the BBB and to GBM cells via targeting, control release at the target site and reduce off-target toxicity [45–50].

They are classified as organic, inorganic and biological carriers with liposomes and polymeric NPs being the most successful among the organic ones and carbon nanotubes (CNTs), and gold NPs (Au-NPs) leading the inorganic category. Given the hyper selectivity of the BBB, however, biomimetic devices such as NPs coated with erythrocyte membranes, showed a better efficacy in overcoming this obstacle [51–53].

We have already discussed active and passive transport through the BBB. Theoretically, NPs could exploit both these mechanisms, however anti-tumor drugs are usually larger molecules, meaning that diffusion-dependent routes are inapplicable. Thus, energy-dependent routes are the delivery mechanism of choice [54–56].

Regarding the dimensions, there is no universal optimal size. Most studies on drug delivery through the BBB use NPs in size from 10 to 100 nm, but factors such as the type of NP, associated surface proteins and physiological functioning of the BBB should be considered [57].

The protein corona (PC) is another fundamental aspect of NPs, functional to its efficiency. It is defined as “the outer layer of deliverables” and it is on its interaction with both the NP and the surrounding environment that drugs delivery depends. The thicker and the stabler the PC, the better the outcome. These two characteristics are correlated to the size and concentration of the NPs [58, 59].

The most investigated surface modification is the one of Poly ethylene glycol-poly lactic acid (PEG-PLA) NPs, due to their long circulating behavior in the blood stream after intravenous or intranasal administration [60]. Besides, PEG and PLA are both materials approved by the Food and Drug Administration, ensuring safety to the studies. However, PEG chains inhibit interaction with cell surfaces, hindering BBB penetration. A solution was found in conjugating it to an activatable low molecular weight protamine (ALMWP) to form a cell penetrating peptides able to enhance the targeted therapy and to penetrate the tumor. Because of all these characteristics, ALMWP-NP was then loaded with anti-tumor agents effective on GBM, for example Paclitaxel (PTX) [61]. PTX interferes with the normal breakdown of microtubules, preventing cell division and leading to cell death [62]. Currently approved formulations of PTX lack the ability to pass through the blood-brain barrier, hence the need to associate it to ALMW-NP.

Another well studied molecule added to NPs surface is transferrin, commonly used to transport iron across the blood-brain barrier. Transferrin receptors are restricted to brain capillaries, rendering them a potential for targeting therapy through their internalization of Tf via receptor-mediated endocytosis [57, 63, 64]. However, a high concentration of endogenous Tf saturates the receptors, making this system far from being ideal. Thus, antibodies with affinity for different epitopes on the Tf receptor are being investigated i.e. OX26 anti-Tf receptor monoclonal antibody, though current in vivo experiments showed that they are not able to mediate actual crossing of the endothelial cell layer, meaning that more studies will be required [65–67].

Melanotransferrin (p97) is a GPI-anchored protein expressed in melanomas very similar to Tf that instead of Tf receptor, are transported through the endothelium by low-density lipoprotein-receptor related protein (LRP). Not only this has proven to be an efficient DDS, but, in contrast with Tf, plasma concentration of endogenous p97 is relatively low, hence does not saturate binding sites [68, 69]. Accordingly, relative to the Tf receptor system, the melanotransferrin-receptor system emerges as a preferred targeting vector for drug transport into the brain.

Other molecules can be used to modify NPs surfaces, including proteins and antibodies targeting the insulin receptor, the low-density lipoprotein receptor and the leptin receptor. However, all these still present challenges related to collateral effect for glucose metabolism, lack of brain capillary endothelium specificity and, regarding the leptin system, an inappropriate indication in obese individuals.

Small molecules were assessed too, nucleoside adenosine for example was considered due to its involvement in neuronal and synaptic function. Adenosine proved to be able to reduce tight junction cohesion and, accordingly, to increase the BBB permeability. However, it also showed an inefficacy in crossing the BBB due to its short circulation time [70–73]. Hence an association of adenosine to squalene NPs to protect it from metabolization was experimented, resulting in an increase of its circulation time [74].

One more promising surface modification is represented by RGD (Arg-Gly-Asp) peptide sequence, a cell adhesion motif found in many extracellular matrix proteins. Its role in cell attachment, migration, and differentiation turned a spotlight on this molecule, leading to interesting results. RGD peptides bind integrin receptors, inhibit tumor migration and angiogenesis, and can also target the delivery of anti-tumor drugs. Recent studies associated RGD peptides with silver NPs (Ag NPs) and nano-selenium (Se NPs). This DDS induced glioma cells ROS production, decreased mitochondrial membrane potential, and caused MAPKs activation, ultimately resulting in tumor cell apoptosis [75, 76].

A comparative summary of the main surface modification strategies, their mechanisms of action, advantages, and limitations is provided in Table 1.

**Table 1:** Summary of Surface-Modified nanoparticle strategies for glioblastoma treatment this table provides a concise overview of key nanoparticle (NP) strategies explored for glioblastoma multiforme (GBM), focusing on their targeting mechanisms, therapeutic benefits, and limitations in clinical translation

Targeting Strategy	Mechanism of Action	Advantages	Limitations
Passive Targeting (EPR effect)	Exploits leaky tumor vasculature to accumulate nanoparticles passively.	Simple design; effective in rodent models.	Limited efficacy in human GBM due to less permeable vasculature.
Ligand-Mediated Targeting	Targets overexpressed receptors on BBB or GBM cells (e.g., transferrin, CD44).	High specificity; reduced off-target effects.	Requires precise receptor characterization; may vary between patients.
Dual-Ligand Functionalization	Combines multiple receptor-targeting ligands for enhanced selectivity.	Enhanced BBB crossing and tumor selectivity.	Complex synthesis and stability concerns.
Stimuli-Responsive Systems	Alters size/charge in response to pH, enzymes, or redox conditions in the tumor.	Improved penetration and release in TME.	Requires accurate tumor microenvironment mapping.
Immunomodulatory NPs	Reprograms tumor-associated macrophages or delivers immune agents to reshape the TME.	Enhances immune response; synergistic with checkpoint inhibitors.	Potential off-target immune activation; requires safety validation.

In parallel with conventional chemical and biological functionalization, recent investigations have explored the potential of green-synthesized nanoparticles in oncology, offering a sustainable and biocompatible alternative for targeted therapies. Green-synthesized nanoparticles have lately attracted interest in nanomedicine because of their excellent biocompatibility, affordability, and potential therapeutic and diagnostic uses. These eco-friendly nanocarriers are being progressively investigated for targeted cancer treatments. Montazersaheb et al. shown that silver nanoparticles derived from pumpkin peel serve as efficient radiosensitizers in triple-negative breast cancer [77]. Rosic et al. examined the function of nanobiomaterials in cancer signaling and gene therapy, highlighting its diagnostic and therapeutic applications [78]. Güneş et al. synthesized silver-coated iron oxide nanoparticles utilizing Hibiscus esculentus, exhibiting antibacterial and magnetic characteristics [79]. Lastly, Keskin et al. (2025) validated the cytotoxic and antibacterial properties of green silver nanoparticles derived from Anchusa officinalis [80].

### Surface Characterization Techniques

As previously discussed, the absence of a universal marker for effective targeting remains a major obstacle in the advancement of NP-based therapies. Compounding this issue is the widespread problem of incomplete nanomaterial characterization, which continues to hinder progress in the field. Although this limitation is well recognized within the scientific community, it remains challenging to address due to several intrinsic factors. Inadequate characterization not only impedes a comprehensive understanding of nanomaterials, but also undermines the reproducibility of experimental results, which is a critical barrier to translating promising in vitro findings into clinical applications [11, 81].

Nonetheless, several surface characterization techniques are currently available. Among those most frequently employed in the Literature we reviewed are zeta potential measurement and X-ray photoelectron spectroscopy. Zeta potential analysis evaluates the electrostatic repulsion between similarly charged particles, providing insight into colloidal stability and surface charge behavior. X-ray photoelectron spectroscopy, a powerful photoemission spectroscopy method, generates electron energy spectra by irradiating materials with X-rays, enabling detailed information on elemental composition and chemical bonding states [82, 83].

It is essential to emphasize the role of surface characterization in the development of NP-based therapies. Since the surface of NPs is the primary interface with biological systems, its properties directly influence the formation of the protein corona, ligand-receptor binding, circulation time, and even the overall toxicity of the nanocarrier system. Thorough surface characterization, therefore, is not a peripheral task but a central component of NP design and translational potential.

### Translational Challenges and Future Directions

Over the last decade, there has been an increased interest towards GBM targeting and considerable advancements have been made in the development of surface-modified NPs, providing them with leadership in this field [11, 84, 85]. Considering the role of BBB in preventing the success of most anti-tumoral therapies, new GBM-targeting studies do not focus only on GBM itself but attempt to improve the rate of BBB crossing as well. However, they still lack specificity within the CNS and can lead to toxic effects to local healthy cells. The only solution would appear to be co-targeting both GBM ligands, to improve selectivity and safety profile, and BBB ligands, to improve the delivery [28, 86, 87]. Dual-targeting daunorubicin Liposomes were developed by conjugating with p-aminophenyl-alpha-D-manno-pyranoside and transferrin for transporting drug across the BBB and then targeting glioma cells. This in vitro study showed an increasement up to 24.9% in the transport ratio through the BBB, confirming the interest towards dual-target therapy in GBM treatment [87]. Another more recent in vitro study developed a pH-sensitive dual-targeting drug carrier, G4-DOX-PEG-Tf-TAM, and conjugated it with Transferrin in the exterior and Tamoxifen in the interior of the fourth generation PAMAM dendrimers. Results exhibited a higher BBB transportation ability, with the transporting ratio of 6.06% in 3 h [88]. One more in vivo study tested a novel dual-targeting liposomal carrier, incorporated with Tamoxifen and conjugated on their surface with wheat germ agglutinin. Topotecan was then loaded in liposomes. Both in vitro and in vivo results showed a beneficial effect and encouraged further developments in this direction [89]. However, finding two ligands, characterizing them and simultaneously testing their interactions still represents a challenge that will require more research and more studies before becoming a consolidated part of GBM therapy.

All these nanocarrier–drug systems, the so called “Trojan horse complexes,” [90, 91] serve as delivery devices that transport specific drugs through the brain endothelium and, subsequently, release it at the appropriate site [66]. This local treatment, compared to systemic ones, would not only improve the efficiency of the anti-tumor drugs, concentrating it right where it is needed, but would also reduce toxicity effects [84]. This does not mean that NPs lack adverse effects. The most common ones are myelotoxicity, vomiting or nausea and Palmoplantar Erythrodysesthesia. Among the rarer, pulmonary embolism, cerebral edema, pneumonia, mucositis and hypophosphatemia represent the most life-threatening ones [92]. It is important to underline that basically no therapeutical gesture in medicine exempt from side effects, including life threatening ones, however, compared to traditional therapies against GBM, NPs seem to have a controlled dose of adverse effects [83] However, longer studies will be required to exclude long terms complications as well carcinogenicity. The possibility that the use of nanomaterials could lead to genetic alterations and nucleic acids abnormalities was confirmed by Zhang et al. and Singh et al., hence this aspect will need further studies in time [84–86].

Despite promising results, challenges remain in translating success from in vitro and animal models to human patients. For example, a Phase II study of PEGylated liposomal doxorubicin with temozolomide and radiotherapy for GBM found the treatment to be feasible and safe but did not meaningfully improve patient outcomes [84]. Most experiments rely on in vitro models or animal studies. Recent research tried to develop a human-relevant GBM models that could be used to investigate the efficiency of NPs in an environment as close as possible to the in vivo human one [97–100]. They represent an efficient temporary solution, substituting animal models and avoiding ethical concerns that would arise in in vivo studies. However, as for now, they still present some limitations related to their inability to recreate the temporal and spatial complexity of GBM that make them impossible to be considered a substitute for human testing [101, 102].

Finally, the challenge still represented by surface characterization prevents further advancements, requiring more studies to understand better these new technologies. Researchers, technicians, and industrialists should cooperate to explore options and usefully exploit nanotechnology in field experiments [103]. More studies will be needed to detect a universal biomarker too, to focus all the efforts on it [104]. This raises the demand for developing smart arrays (interdigitated electrode system), multiple detection methods, highly sensitive transducers, and microfluidic systems [105–111].

Alongside systemic administration, other locoregional techniques have emerged to improve nanoparticle delivery in glioblastoma, especially in surmounting the limiting characteristics of the blood-brain barrier. Convection-enhanced delivery (CED) facilitates the direct injection of therapeutic drugs into the brain parenchyma, enabling regulated distribution while circumventing systemic circulation. Preclinical and early clinical investigations indicate that CED markedly enhances drug distribution within the tumor and adjacent infiltrative margin, while reducing systemic toxicity [11]. The intrathecal and intratumoral methods offer potential benefits, especially for confined or recurrent glioblastomas. Although these techniques necessitate neurosurgical intervention and entail procedural risks, they provide potential for attaining therapeutic concentrations that systemic administration frequently does not achieve. Comparative assessments of systemic and intratumoral delivery are crucial for establishing the best therapeutic index and enhancing the clinical translation of nanoparticle-based treatments in GBM patients.

### Regulatory and Translational Hurdles

Despite considerable preclinical advancements in the development of nanoparticle platforms for glioblastoma treatment, their translation into clinical practice is hindered by other substantial non-biological obstacles. Thee regulatory approval necessitates extensive data on biodistribution, clearance, long-term toxicity, and interactions with standard-of-care therapies. The regulatory framework is further complicated by the combined diagnostic and therapeutic (theranostic) functions of certain nanosystems, which may activate combination product paths under FDA or EMA regulations.

Clinical studies are essential in closing this translational gap, offering data on pharmacokinetics, tolerability, and therapeutic efficacy in human participants. Several current trials are assessing nanoparticle-based delivery systems in individuals with recurrent or treatment-resistant glioblastoma. Table 2 summarized selected trials, demonstrating the variety of nanocarrier techniques being explored and their translational significance.

**Table 2:** Selected ongoing clinical trials of nanoparticle-based strategies in glioblastoma

Clinical Trial Identifier	Intervention Name & Description	Phase	Objective	Translational Relevance
NCT02340156	**Nanoliposomal Irinotecan (Nal-IRI)**: lipid-based carrier for CPT-11 in recurrent GBM	I/II	Assess safety, pharmacokinetics, and dose-limiting toxicity	Demonstrates feasibility of lipid nanocarriers for chemotherapeutic delivery in human GBM
NCT03603379	**BIND-014**: PSMA-targeted polymeric nanoparticle delivering docetaxel	II	Evaluate targeting efficacy and imaging capability	Represents theranostic approach enabling real-time tracking of drug distribution and response

While factors including resabsorption, organ-level accumulation, and nanoparticle clearance are crucial for long-term safety, a comprehensive pharmacokinetic analysis exceeds the purview of this review and has been thoroughly explored in pertinent dedicated literature.

### Combination Strategies

Theranostic nanoparticles signify a notable progression in glioblastoma (GBM) treatment, representing a novel research frontier, that integrates diagnostic and therapeutic capabilities inside a singular nanoplatform.

Theranostic nanoparticles are designed to integrate imaging agents including SPIONs (for MRI), radiolabeled tracers (for PET), and NIR fluorophores (for optical imaging), facilitating real-time, non-invasive observation of biodistribution, tumor targeting, and treatment effectiveness [112, 113]. Enzyme-responsive carriers augment imaging capabilities and facilitate drug release in the GBM microenvironment [112]. Dual-modality technologies integrating MRI and fluorescence facilitate tumor identification during surgical procedures and subsequent monitoring [113]. Theranostic technologies enhance early evaluation of treatment response and increase precision neuro-oncology by the integration of targeted delivery, multimodal imaging, and on-demand release [114].

In addition, the incorporation of nanoparticles (NPs) with traditional treatment methods including radiotherapy, hyperthermia, and phototherapy is a potent translational strategy to improve therapeutic effectiveness in glioblastoma. Gold and hafnium oxide nanoparticles exhibit radiosensitizing properties by enhancing radiation-induced DNA damage and increasing the production of reactive oxygen species, consequently reducing the radiation dose required for tumor management [115, 116]. Likewise, superparamagnetic iron oxide nanoparticles (SPIONs) are under investigation for magnetic hyperthermia, wherein alternating magnetic fields generate localized heating of tumors and impair cellular activity [117]. Photothermal and photodynamic therapies (PTT/PDT) are under investigation, utilizing NIR-absorbing nanoparticles linked with photosensitizers to selectively produce thermal ablation or oxidative stress inside the tumor microenvironment [118]. These combination strategies enhance the synergistic interplay between targeted nanodelivery and conventional treatments to address tumor heterogeneity and resistance mechanisms. Numerous preclinical and early-phase clinical investigations have demonstrated that nanoparticle-mediated radiosensitization or photothermal enhancement may potentially improve survival minimizing collateral tissue damage [119, 120]. The integration of multimodal nanocarriers with tumor-specific ligands or immune modulators may significantly improve tumor specificity and immune activation [121]. As these techniques progress towards clinical application, ongoing investigations persist in refining their safety, biodistribution, and dosimetric profiles in neuro-oncology contexts [122].

## Conclusions

Nanocarrier-based drug delivery systems offer promising strategies for overcoming the BBB and achieving targeted treatment of GBM. Surface-modified NPs, particularly those utilizing dual-targeting approaches, enhance drug delivery efficacy while minimizing off-target toxicity. However, clinical translation remains limited due to challenges related to targeting specificity, safety, and complex ligand interactions. Advancing GBM therapy will require deeper insights into NP surface properties and the identification of universal biomarkers. Interdisciplinary collaboration will be essential to bridge the gap between preclinical success and clinical application.

Future advancements in this domain will necessitate a collaborative and multidisciplinary approach. The integration of nanocarrier-based delivery with both current and emerging treatment options, may produce synergic survival advantages. The advancement of theranostic nanoplatforms that can concurrently administer medications and provide real time imaging may significantly enhance patient monitoring and personalized treatment strategies.

## Electronic Supplementary Material

Below is the link to the electronic supplementary material.

corrected REF List: unable to modify on line

corrected proofs with references and citations in main tex: unable to load

## Abbreviations

Ag-NPs: Silver Nanoparticles
ALMWP: Activatable Low Molecular Weight Protamine
Au-NPs: Gold Nanoparticles
BBB: Blood Brain Barrier
CPP: Cell Penetrating Peptides
CNTs: Carbon Nanotubes
CNS: Central Nervous System
DDS: Drug Delivery System
EGF: Epidermal Growth Factor
EPR: Enhanced Permeation and Retention
FDA: Food and Drug Administration
GBM: Glioblastoma Multiforme
GSCs: Glioblastoma Stem Cells
HIF: Hypoxia-Inducible-Factor
Lf: Lactoferrin
LDL: Low-Density Lipoprotein
LRP: Lipoprotein-Receptor Related Protein
MMP: Matrix Metalloproteases
NPs: Nanoparticles
PEG-PLA: Poly ethylene glycol-poly lactic acid
PC: Protein Corona
PTX: Paclitaxel
RGD: Arg-Gly-Asp
Se-NPs: Selenium Nanoparticles
TAMs: Tumor Associated Macrophages
TANs: Tumor-Associated Nerves
TME: Tumor Microenvironment
Tf: Transferrin
VEGF: Vascular Endothelial Growth Factor
WHO: World Health Organization
