# Supplementary material for: Advancing glioblastoma therapy with surface-modified nanoparticles
Source: Neurol Sci. 2025 Sep 13;46(11):5757–70. doi: 10.1007/s10072-025-08457-4 (PMC12537612; doi:10.1007/s10072-025-08457-4)
Supplement: Supplementary file 1 — corrected REF List: unable to modify on line [file 10072_2025_8457_MOESM1_ESM.docx]

1. Wirsching, H. G., Galanis, E., & Weller, M. (2016). Glioblastoma. *Handbook of clinical neurology*, *134*, 381–397. <https://doi.org/10.1016/B978-0-12-802997-8.00023-2>
2. Gimple, R. C., Bhargava, S., Dixit, D., & Rich, J. N. (2019). Glioblastoma stem cells: lessons from the tumor hierarchy in a lethal cancer. *Genes & development*, *33*(11-12), 591–609. <https://doi.org/10.1101/gad.324301.119>
3. Tan, A. C., Ashley, D. M., López, G. Y., Malinzak, M., Friedman, H. S., & Khasraw, M. (2020). Management of glioblastoma: State of the art and future directions. *CA: a cancer journal for clinicians*, *70*(4), 299–312. <https://doi.org/10.3322/caac.21613>
4. Delgado-López, P. D., & Corrales-García, E. M. (2016). Survival in glioblastoma: a review on the impact of treatment modalities. *Clinical & translational oncology : official publication of the Federation of Spanish Oncology Societies and of the National Cancer Institute of Mexico*, *18*(11), 1062–1071. <https://doi.org/10.1007/s12094-016-1497-x>
5. Louis, D. N., Perry, A., Wesseling, P., Brat, D. J., Cree, I. A., Figarella-Branger, D., Hawkins, C., Ng, H. K., Pfister, S. M., Reifenberger, G., Soffietti, R., von Deimling, A., & Ellison, D. W. (2021). The 2021 WHO Classification of Tumors of the Central Nervous System: a summary. *Neuro-oncology*, *23*(8), 1231–1251. <https://doi.org/10.1093/neuonc/noab106>
6. Smith, H. L., Wadhwani, N., & Horbinski, C. (2022). Major Features of the 2021 WHO Classification of CNS Tumors. *Neurotherapeutics : the journal of the American Society for Experimental NeuroTherapeutics*, *19*(6), 1691–1704. <https://doi.org/10.1007/s13311-022-01249-0>
7. Stupp, R., Mason, W. P., van den Bent, M. J., Weller, M., Fisher, B., Taphoorn, M. J., Belanger, K., Brandes, A. A., Marosi, C., Bogdahn, U., Curschmann, J., Janzer, R. C., Ludwin, S. K., Gorlia, T., Allgeier, A., Lacombe, D., Cairncross, J. G., Eisenhauer, E., Mirimanoff, R. O., European Organisation for Research and Treatment of Cancer Brain Tumor and Radiotherapy Groups, … National Cancer Institute of Canada Clinical Trials Group (2005). Radiotherapy plus concomitant and adjuvant temozolomide for glioblastoma. *The New England journal of medicine*, *352*(10), 987–996. <https://doi.org/10.1056/NEJMoa043330>
8. Lakomy, R., Kazda, T., Selingerova, I., Poprach, A., Pospisil, P., Belanova, R., Fadrus, P., Vybihal, V., Smrcka, M., Jancalek, R., Hynkova, L., Muckova, K., Hendrych, M., Sana, J., Slaby, O., & Slampa, P. (2020). Real-World Evidence in Glioblastoma: Stupp's Regimen After a Decade. *Frontiers in oncology*, *10*, 840. <https://doi.org/10.3389/fonc.2020.00840>
9. Jain K. K. (2018). A Critical Overview of Targeted Therapies for Glioblastoma. *Frontiers in oncology*, *8*, 419. <https://doi.org/10.3389/fonc.2018.00419>
10. Manini, I., Caponnetto, F., Dalla, E., Ius, T., Della Pepa, G. M., Pegolo, E., Bartolini, A., La Rocca, G., Menna, G., Di Loreto, C., Olivi, A., Skrap, M., Sabatino, G., & Cesselli, D. (2020). Heterogeneity Matters: Different Regions of Glioblastoma Are Characterized by Distinctive Tumor-Supporting Pathways. *Cancers*, *12*(10), 2960. <https://doi.org/10.3390/cancers12102960>
11. Ius, T., Somma, T., Pasqualetti, F., Berardinelli, J., Vitulli, F., Caccese, M., Cella, E., Cenciarelli, C., Pozzoli, G., Sconocchia, G., Zeppieri, M., Gerardo, C., Caffo, M., & Lombardi, G. (2024). Local therapy in glioma: An evolving paradigm from history to horizons (Review). *Oncology letters*, *28*(3), 440. <https://doi.org/10.3892/ol.2024.14573>
12. Ius, T., Sabatino, G., Panciani, P. P., Fontanella, M. M., Rudà, R., Castellano, A., Barbagallo, G. M. V., Belotti, F., Boccaletti, R., Catapano, G., Costantino, G., Della Puppa, A., Di Meco, F., Gagliardi, F., Garbossa, D., Germanò, A. F., Iacoangeli, M., Mortini, P., Olivi, A., Pessina, F., … Esposito, V. (2023). Surgical management of Glioma Grade 4: technical update from the neuro-oncology section of the Italian Society of Neurosurgery (SINch®): a systematic review. *Journal of neuro-oncology*, *162*(2), 267–293. <https://doi.org/10.1007/s11060-023-04274-x>
13. Ballabh, P., Braun, A., & Nedergaard, M. (2004). The blood-brain barrier: an overview: structure, regulation, and clinical implications. *Neurobiology of disease*, *16*(1), 1–13. <https://doi.org/10.1016/j.nbd.2003.12.016>
14. Daneman, R., & Prat, A. (2015). The blood-brain barrier. *Cold Spring Harbor perspectives in biology*, *7*(1), a020412. <https://doi.org/10.1101/cshperspect.a020412>
15. Dixit, S., Novak, T., Miller, K., Zhu, Y., Kenney, M. E., & Broome, A. M. (2015). Transferrin receptor-targeted theranostic gold nanoparticles for photosensitizer delivery in brain tumors. *Nanoscale*, *7*(5), 1782–1790. <https://doi.org/10.1039/c4nr04853a>
16. Ulbrich, K., Knobloch, T., & Kreuter, J. (2011). Targeting the insulin receptor: nanoparticles for drug delivery across the blood-brain barrier (BBB). *Journal of drug targeting*, *19*(2), 125–132. <https://doi.org/10.3109/10611861003734001>
17. He, Chunsheng & Li, Jason & Cai, Ping & Ahmed, Taksim & Henderson, Jeffrey & Foltz, Warren & Bendayan, Reina & Rauth, Andrew & Wu, Xiao Yu. (2018). Two-Step Targeted Hybrid Nanoconstructs Increase Brain Penetration and Efficacy of the Therapeutic Antibody Trastuzumab against Brain Metastasis of HER2-Positive Breast Cancer. Advanced Functional Materials. 28. 1705668. <https://doi.org/10.1002/adfm.201705668>
18. Qiao, R., Jia, Q., Hüwel, S., Xia, R., Liu, T., Gao, F., Galla, H. J., & Gao, M. (2012). Receptor-mediated delivery of magnetic nanoparticles across the blood-brain barrier. *ACS nano*, *6*(4), 3304–3310. <https://doi.org/10.1021/nn300240p>
19. Wei, X., Zhan, C., Shen, Q., Fu, W., Xie, C., Gao, J., Peng, C., Zheng, P., & Lu, W. (2015). A D-peptide ligand of nicotine acetylcholine receptors for brain-targeted drug delivery. *Angewandte Chemie (International ed. in English)*, *54*(10), 3023–3027. <https://doi.org/10.1002/anie.201411226>
20. Jiang, X., Xin, H., Ren, Q., Gu, J., Zhu, L., Du, F., Feng, C., Xie, Y., Sha, X., & Fang, X. (2014). Nanoparticles of 2-deoxy-D-glucose functionalized poly(ethylene glycol)-co-poly(trimethylene carbonate) for dual-targeted drug delivery in glioma treatment. *Biomaterials*, *35*(1), 518–529. <https://doi.org/10.1016/j.biomaterials.2013.09.094>
21. Li, J., Guo, Y., Kuang, Y., An, S., Ma, H., & Jiang, C. (2013). Choline transporter-targeting and co-delivery system for glioma therapy. *Biomaterials*, *34*(36), 9142–9148. <https://doi.org/10.1016/j.biomaterials.2013.08.030>
22. Martins, C., & Sarmento, B. (2023). Multi-ligand functionalized blood-to-tumor sequential targeting strategies in the field of glioblastoma nanomedicine. *Wiley interdisciplinary reviews. Nanomedicine and nanobiotechnology*, *15*(5), e1893. <https://doi.org/10.1002/wnan.1893>
23. Gao, J. Q., Lv, Q., Li, L. M., Tang, X. J., Li, F. Z., Hu, Y. L., & Han, M. (2013). Glioma targeting and blood-brain barrier penetration by dual-targeting doxorubincin liposomes. *Biomaterials*, *34*(22), 5628–5639. <https://doi.org/10.1016/j.biomaterials.2013.03.097>
24. Dotiwala, A. K., McCausland, C., & Samra, N. S. (2023). Anatomy, Head and Neck: Blood Brain Barrier. In *StatPearls*. StatPearls Publishing.
25. Weathers, S. P., & de Groot, J. (2015). VEGF Manipulation in Glioblastoma. *Oncology (Williston Park, N.Y.)*, *29*(10), 720–727.
26. Yi, Y., Hsieh, I. Y., Huang, X., Li, J., & Zhao, W. (2016). Glioblastoma Stem-Like Cells: Characteristics, Microenvironment, and Therapy. *Frontiers in pharmacology*, *7*, 477. <https://doi.org/10.3389/fphar.2016.00477>
27. Pavon, L. F., Marti, L. C., Sibov, T. T., Malheiros, S. M., Brandt, R. A., Cavalheiro, S., & Gamarra, L. F. (2014). In vitro Analysis of Neurospheres Derived from Glioblastoma Primary Culture: A Novel Methodology Paradigm. *Frontiers in neurology*, *4*, 214. <https://doi.org/10.3389/fneur.2013.00214>
28. Liebelt, B. D., Shingu, T., Zhou, X., Ren, J., Shin, S. A., & Hu, J. (2016). Glioma Stem Cells: Signaling, Microenvironment, and Therapy. *Stem cells international*, *2016*, 7849890. <https://doi.org/10.1155/2016/7849890>
29. Brescia, P., Ortensi, B., Fornasari, L., Levi, D., Broggi, G., & Pelicci, G. (2013). CD133 is essential for glioblastoma stem cell maintenance. *Stem cells (Dayton, Ohio)*, *31*(5), 857–869. <https://doi.org/10.1002/stem.1317>
30. Shibahara, I., Sonoda, Y., Saito, R., Kanamori, M., Yamashita, Y., Kumabe, T., Watanabe, M., Suzuki, H., Watanabe, T., Ishioka, C., & Tominaga, T. (2013). The expression status of CD133 is associated with the pattern and timing of primary glioblastoma recurrence. *Neuro-oncology*, *15*(9), 1151–1159. <https://doi.org/10.1093/neuonc/not066>
31. Han, M., Guo, L., Zhang, Y., Huang, B., Chen, A., Chen, W., Liu, X., Sun, S., Wang, K., Liu, A., & Li, X. (2016). Clinicopathological and Prognostic Significance of CD133 in Glioma Patients: A Meta-Analysis. *Molecular neurobiology*, *53*(1), 720–727. <https://doi.org/10.1007/s12035-014-9018-9>
32. Yan, X., Ma, L., Yi, D., Yoon, J. G., Diercks, A., Foltz, G., Price, N. D., Hood, L. E., & Tian, Q. (2011). A CD133-related gene expression signature identifies an aggressive glioblastoma subtype with excessive mutations. *Proceedings of the National Academy of Sciences of the United States of America*, *108*(4), 1591–1596. <https://doi.org/10.1073/pnas.1018696108>
33. Nayak, Aadya & Warrier, Neerada & Raman, Rachana & Prabhu, Vijendra & Kumar, Praveen. (2024). Targeted Delivery of Nanomedicines to Glioblastoma: Overcoming the Clinical Barrier. Journal of Drug Delivery Science and Technology. 99. 105980. <https://doi.org/10.1016/j.jddst.2024.105980>
34. Mao, J. M., Liu, J., Guo, G., Mao, X. G., & Li, C. X. (2015). Glioblastoma vasculogenic mimicry: signaling pathways progression and potential anti-angiogenesis targets. *Biomarker research*, *3*, 8. <https://doi.org/10.1186/s40364-015-0034-3>
35. Shi, Y., van der Meel, R., Chen, X., & Lammers, T. (2020). The EPR effect and beyond: Strategies to improve tumor targeting and cancer nanomedicine treatment efficacy. *Theranostics*, *10*(17), 7921–7924. <https://doi.org/10.7150/thno.49577>
36. Danhier F. (2016). To exploit the tumor microenvironment: Since the EPR effect fails in the clinic, what is the future of nanomedicine?. *Journal of controlled release : official journal of the Controlled Release Society*, *244*(Pt A), 108–121. <https://doi.org/10.1016/j.jconrel.2016.11.015>
37. Nakamura, Y., Mochida, A., Choyke, P. L., & Kobayashi, H. (2016). Nanodrug Delivery: Is the Enhanced Permeability and Retention Effect Sufficient for Curing Cancer?. *Bioconjugate chemistry*, *27*(10), 2225–2238. <https://doi.org/10.1021/acs.bioconjchem.6b00437>
38. de Lázaro, I., & Mooney, D. J. (2020). A nanoparticle's pathway into tumours. *Nature materials*, *19*(5), 486–487. <https://doi.org/10.1038/s41563-020-0669-9>
39. Golombek, S. K., May, J. N., Theek, B., Appold, L., Drude, N., Kiessling, F., & Lammers, T. (2018). Tumor targeting via EPR: Strategies to enhance patient responses. *Advanced drug delivery reviews*, *130*, 17–38. <https://doi.org/10.1016/j.addr.2018.07.007>
40. Lv, Y., Xu, C., Zhao, X., Lin, C., Yang, X., Xin, X., Zhang, L., Qin, C., Han, X., Yang, L., He, W., & Yin, L. (2018). Nanoplatform Assembled from a CD44-Targeted Prodrug and Smart Liposomes for Dual Targeting of Tumor Microenvironment and Cancer Cells. *ACS nano*, *12*(2), 1519–1536. <https://doi.org/10.1021/acsnano.7b08051>
41. Ambasta, R. K., Sharma, A., & Kumar, P. (2011). Nanoparticle mediated targeting of VEGFR and cancer stem cells for cancer therapy. *Vascular cell*, *3*, 26. <https://doi.org/10.1186/2045-824X-3-26>
42. Gysler, S. M., & Drapkin, R. (2021). Tumor innervation: peripheral nerves take control of the tumor microenvironment. *The Journal of clinical investigation*, *131*(11), e147276. <https://doi.org/10.1172/JCI147276>
43. Zahalka, A. H., Arnal-Estapé, A., Maryanovich, M., Nakahara, F., Cruz, C. D., Finley, L. W. S., & Frenette, P. S. (2017). Adrenergic nerves activate an angio-metabolic switch in prostate cancer. *Science (New York, N.Y.)*, *358*(6361), 321–326. <https://doi.org/10.1126/science.aah5072>
44. Ahlawat, J., Guillama Barroso, G., Masoudi Asil, S., Alvarado, M., Armendariz, I., Bernal, J., Carabaza, X., Chavez, S., Cruz, P., Escalante, V., Estorga, S., Fernandez, D., Lozano, C., Marrufo, M., Ahmad, N., Negrete, S., Olvera, K., Parada, X., Portillo, B., Ramirez, A., … Narayan, M. (2020). Nanocarriers as Potential Drug Delivery Candidates for Overcoming the Blood-Brain Barrier: Challenges and Possibilities. *ACS omega*, *5*(22), 12583–12595. <https://doi.org/10.1021/acsomega.0c01592>
45. Farooq, M., Scalia, G., Umana, G. E., Parekh, U. A., Naeem, F., Abid, S. F., Khan, M. H., Zahra, S. G., Sarkar, H. P., & Chaurasia, B. (2023). A Systematic Review of Nanomedicine in Glioblastoma Treatment: Clinical Efficacy, Safety, and Future Directions. *Brain sciences*, *13*(12), 1727. <https://doi.org/10.3390/brainsci13121727>
46. Khan, I., Baig, M. H., Mahfooz, S., Imran, M. A., Khan, M. I., Dong, J. J., Cho, J. Y., & Hatiboglu, M. A. (2022). Nanomedicine for glioblastoma: Progress and future prospects. *Seminars in cancer biology*, *86*(Pt 2), 172–186. <https://doi.org/10.1016/j.semcancer.2022.06.007>
47. Dang, Y., & Guan, J. (2020). Nanoparticle-based drug delivery systems for cancer therapy. *Smart materials in medicine*, *1*, 10–19. <https://doi.org/10.1016/j.smaim.2020.04.001>
48. Anselmo, A. C., & Mitragotri, S. (2016). Nanoparticles in the clinic. *Bioengineering & translational medicine*, *1*(1), 10–29. <https://doi.org/10.1002/btm2.10003>
49. Amaral, M., Cruz, N., Rosa, A., Nogueira, B., Costa, D., Santos, F., Brazão, M., Policarpo, P., Mateus, R., Kobozev, Y., & Reis, C. P. (2021). An update of advanced nanoplatforms for Glioblastoma Multiforme Management. *EXCLI journal*, *20*, 1544–1570. <https://doi.org/10.17179/excli2021-4393>
50. Di Filippo, L. D., Duarte, J. L., Luiz, M. T., de Araújo, J. T. C., & Chorilli, M. (2021). Drug Delivery Nanosystems in Glioblastoma Multiforme Treatment: Current State of the Art. *Current neuropharmacology*, *19*(6), 787–812. <https://doi.org/10.2174/1570159X18666200831160627>
51. Liu, D., Dai, X., Ye, L., Wang, H., Qian, H., Cheng, H., & Wang, X. (2023). Nanotechnology meets glioblastoma multiforme: Emerging therapeutic strategies. *Wiley interdisciplinary reviews. Nanomedicine and nanobiotechnology*, *15*(1), e1838. <https://doi.org/10.1002/wnan.1838>
52. Zhang, N., Wei, L., Ye, M., Kang, C., & You, H. (2020). Treatment Progress of Immune Checkpoint Blockade Therapy for Glioblastoma. *Frontiers in immunology*, *11*, 592612. <https://doi.org/10.3389/fimmu.2020.592612>
53. Wang, S., Yang, L., He, W., Zheng, M., & Zou, Y. (2025). Cell Membrane Camouflaged Biomimetic Nanoparticles as a Versatile Platform for Brain Diseases Treatment. *Small methods*, *9*(1), e2400096. <https://doi.org/10.1002/smtd.202400096>
54. Jena, L., McErlean, E., & McCarthy, H. (2020). Delivery across the blood-brain barrier: nanomedicine for glioblastoma multiforme. *Drug delivery and translational research*, *10*(2), 304–318. <https://doi.org/10.1007/s13346-019-00679-2>
55. Li, J., Zhao, J., Tan, T., Liu, M., Zeng, Z., Zeng, Y., Zhang, L., Fu, C., Chen, D., & Xie, T. (2020). Nanoparticle Drug Delivery System for Glioma and Its Efficacy Improvement Strategies: A Comprehensive Review. *International journal of nanomedicine*, *15*, 2563–2582. <https://doi.org/10.2147/IJN.S243223>
56. Liu, Z., Ji, X., He, D., Zhang, R., Liu, Q., & Xin, T. (2022). Nanoscale Drug Delivery Systems in Glioblastoma. *Nanoscale research letters*, *17*(1), 27. <https://doi.org/10.1186/s11671-022-03668-6>
57. Hersh, A. M., Alomari, S., & Tyler, B. M. (2022). Crossing the Blood-Brain Barrier: Advances in Nanoparticle Technology for Drug Delivery in Neuro-Oncology. *International journal of molecular sciences*, *23*(8), 4153. <https://doi.org/10.3390/ijms23084153>
58. Treuel, L., & Nienhaus, G. U. (2012). Toward a molecular understanding of nanoparticle-protein interactions. *Biophysical reviews*, *4*(2), 137–147. <https://doi.org/10.1007/s12551-012-0072-0>
59. Huang, W., Xiao, G., Zhang, Y., & Min, W. (2021). Research progress and application opportunities of nanoparticle-protein corona complexes. *Biomedicine & pharmacotherapy = Biomedecine & pharmacotherapie*, *139*, 111541. <https://doi.org/10.1016/j.biopha.2021.111541>
60. Gao, X., Tao, W., Lu, W., Zhang, Q., Zhang, Y., Jiang, X., & Fu, S. (2006). Lectin-conjugated PEG-PLA nanoparticles: preparation and brain delivery after intranasal administration. *Biomaterials*, *27*(18), 3482–3490. <https://doi.org/10.1016/j.biomaterials.2006.01.038>
61. Gu, G., Xia, H., Hu, Q., Liu, Z., Jiang, M., Kang, T., Miao, D., Tu, Y., Pang, Z., Song, Q., Yao, L., Chen, H., Gao, X., & Chen, J. (2013). PEG-co-PCL nanoparticles modified with MMP-2/9 activatable low molecular weight protamine for enhanced targeted glioblastoma therapy. *Biomaterials*, *34*(1), 196–208. <https://doi.org/10.1016/j.biomaterials.2012.09.044>
62. AbdEl-Haq, M., Kumar, A., Ait Mohand, F. E., Kravchenko-Balasha, N., Rottenberg, Y., & Domb, A. J. (2023). Paclitaxel Delivery to the Brain for Glioblastoma Treatment. *International journal of molecular sciences*, *24*(14), 11722. <https://doi.org/10.3390/ijms241411722>
63. Visser, C. C., Stevanović, S., Heleen Voorwinden, L., Gaillard, P. J., Crommelin, D. J., Danhof, M., & De Boer, A. G. (2004). Validation of the transferrin receptor for drug targeting to brain capillary endothelial cells in vitro. *Journal of drug targeting*, *12*(3), 145–150. <https://doi.org/10.1080/10611860410001701706>
64. Ulbrich, K., Hekmatara, T., Herbert, E., & Kreuter, J. (2009). Transferrin- and transferrin-receptor-antibody-modified nanoparticles enable drug delivery across the blood-brain barrier (BBB). *European journal of pharmaceutics and biopharmaceutics : official journal of Arbeitsgemeinschaft fur Pharmazeutische Verfahrenstechnik e.V*, *71*(2), 251–256. <https://doi.org/10.1016/j.ejpb.2008.08.021>
65. Paris-Robidas, S., Emond, V., Tremblay, C., Soulet, D., & Calon, F. (2011). In vivo labeling of brain capillary endothelial cells after intravenous injection of monoclonal antibodies targeting the transferrin receptor. *Molecular pharmacology*, *80*(1), 32–39. <https://doi.org/10.1124/mol.111.071027>
66. Moos, T., & Morgan, E. H. (2001). Restricted transport of anti-transferrin receptor antibody (OX26) through the blood-brain barrier in the rat. *Journal of neurochemistry*, *79*(1), 119–129. <https://doi.org/10.1046/j.1471-4159.2001.00541.x>
67. Gosk, S., Vermehren, C., Storm, G., & Moos, T. (2004). Targeting anti-transferrin receptor antibody (OX26) and OX26-conjugated liposomes to brain capillary endothelial cells using in situ perfusion. *Journal of cerebral blood flow and metabolism : official journal of the International Society of Cerebral Blood Flow and Metabolism*, *24*(11), 1193–1204. <https://doi.org/10.1097/01.WCB.0000135592.28823.47>
68. Georgieva, J. V., Hoekstra, D., & Zuhorn, I. S. (2014). Smuggling Drugs into the Brain: An Overview of Ligands Targeting Transcytosis for Drug Delivery across the Blood-Brain Barrier. *Pharmaceutics*, *6*(4), 557–583. <https://doi.org/10.3390/pharmaceutics6040557>
69. Demeule, M., Poirier, J., Jodoin, J., Bertrand, Y., Desrosiers, R. R., Dagenais, C., Nguyen, T., Lanthier, J., Gabathuler, R., Kennard, M., Jefferies, W. A., Karkan, D., Tsai, S., Fenart, L., Cecchelli, R., & Béliveau, R. (2002). High transcytosis of melanotransferrin (P97) across the blood-brain barrier. *Journal of neurochemistry*, *83*(4), 924–933. <https://doi.org/10.1046/j.1471-4159.2002.01201.x>
70. Boison D. (2008). Adenosine as a neuromodulator in neurological diseases. *Current opinion in pharmacology*, *8*(1), 2–7. <https://doi.org/10.1016/j.coph.2007.09.002>
71. Stone, T. W., Ceruti, S., & Abbracchio, M. P. (2009). Adenosine receptors and neurological disease: neuroprotection and neurodegeneration. *Handbook of experimental pharmacology*, (193), 535–587. <https://doi.org/10.1007/978-3-540-89615-9_17>
72. Möser, G. H., Schrader, J., & Deussen, A. (1989). Turnover of adenosine in plasma of human and dog blood. *The American journal of physiology*, *256*(4 Pt 1), C799–C806. <https://doi.org/10.1152/ajpcell.1989.256.4.C799>
73. Gao, X., Qian, J., Zheng, S., Changyi, Y., Zhang, J., Ju, S., Zhu, J., & Li, C. (2014). Overcoming the blood-brain barrier for delivering drugs into the brain by using adenosine receptor nanoagonist. *ACS nano*, *8*(4), 3678–3689. <https://doi.org/10.1021/nn5003375>
74. Gaudin, A., Yemisci, M., Eroglu, H., Lepetre-Mouelhi, S., Turkoglu, O. F., Dönmez-Demir, B., Caban, S., Sargon, M. F., Garcia-Argote, S., Pieters, G., Loreau, O., Rousseau, B., Tagit, O., Hildebrandt, N., Le Dantec, Y., Mougin, J., Valetti, S., Chacun, H., Nicolas, V., Desmaële, D., … Couvreur, P. (2014). Squalenoyl adenosine nanoparticles provide neuroprotection after stroke and spinal cord injury. *Nature nanotechnology*, *9*(12), 1054–1062. <https://doi.org/10.1038/nnano.2014.274>
75. Liu, W., Su, J., Shi, Q., Wang, J., Chen, X., Zhang, S., Li, M., Cui, J., Fan, C., Sun, B., & Wang, G. (2021). RGD Peptide-Conjugated Selenium Nanocomposite Inhibits Human Glioma Growth by Triggering Mitochondrial Dysfunction and ROS-Dependent MAPKs Activation. *Frontiers in bioengineering and biotechnology*, *9*, 781608. <https://doi.org/10.3389/fbioe.2021.781608>
76. Hosseinikhah, Seyedeh Maryam & Vahdat lasemi, Fatemeh & Farhoudi, Leila & Gupta, Garima & Kesharwani, Prashant & Sahebkar, Amirhossein. (2024). RGD-decorated nanoparticles: Therapeutic potential beyond cancer. Journal of Drug Delivery Science and Technology. 98. 105924. <https://doi.org/10.1016/j.jddst.2024.105924>
77. Montazersaheb, Soheila & Eftekhari, Aziz & Shafaroodi, Amir & Tavakoli, Soodeh & Jafari, Sara & Baran, Ayşe & Baran, Mehmet & Jafari, Sevda & Ahmadian, Elham. (2024). Green-synthesized silver nanoparticles from peel extract of pumpkin as a potent radiosensitizer against triple-negative breast cancer (TNBC). Cancer Nanotechnology. 15. <https://doi.org/10.1186/s12645-024-00285-z>
78. Rosic, Gvozden. (2024). Cancer signaling, cell/gene therapy, diagnosis and role of nanobiomaterials. Advances in Biology & Earth Sciences. 9. 11-34. <https://doi.org/10.62476/abes9s11>
79. Gunes, Muslum & Ertaş, Erdal & Seyhmus, Tumur & Zulfugarova, Parvin & Nuriyeva, Fidan & Kavetskyy, Taras & Kukhazh, Yuliia & Grozdov, Pavlo & Šauša, O. & Smutok, Oleh & Ganbarov, Dashgin & Kiv, Arnold. (2025). Synthesis and Antibacterial Evaluation of Silver-Coated Magnetic Iron Oxide/Activated Carbon Nanoparticles Derived from Hibiscus esculentus. [DOI:10.20944/preprints202505.1245.v1](https://www.preprints.org/manuscript/202505.1245/v1)
80. Keskin C, Aslan S, Baran MF, Baran A, Eftekhari A, Adıcan MT, Ahmadian E, Arslan S, Mohamed AJ. Green Synthesis and Characterization of Silver Nanoparticles Using *Anchusa Officinalis*: Antimicrobial and Cytotoxic Potential. Int J Nanomedicine. 2025 Apr 12;20:4481-4502. doi: 10.2147/IJN.S511217.
81. Baer, D. R., Engelhard, M. H., Johnson, G. E., Laskin, J., Lai, J., Mueller, K., Munusamy, P., Thevuthasan, S., Wang, H., Washton, N., Elder, A., Baisch, B. L., Karakoti, A., Kuchibhatla, S. V., & Moon, D. (2013). Surface characterization of nanomaterials and nanoparticles: Important needs and challenging opportunities. *Journal of vacuum science & technology. A, Vacuum, surfaces, and films : an official journal of the American Vacuum Society*, *31*(5), 50820. <https://doi.org/10.1116/1.4818423>
82. Bekas, Dimitrios & Tsirka, Kyriaki & Baltzis, Dimitris & Paipetis, Alkiviadis. (2015). Self-healing materials: A review of advances in materials, evaluation, characterization and monitoring techniques. Composites Part B: Engineering. 87. <https://doi.org/10.1016/j.compositesb.2015.09.057>
83. Krishna, Yathin & Fauzan, Ir Ts Dr Mohd Faizal & Saidur, R. & Ng, K.C. & Aslfattahi, Navid. (2020). State-of-the-art heat transfer fluids for parabolic trough collector. International Journal of Heat and Mass Transfer. 152. 119541. <https://doi.org/10.1016/j.ijheatmasstransfer.2020.119541>
84. Qian, L., Zheng, J., Wang, K., Tang, Y., Zhang, X., Zhang, H., Huang, F., Pei, Y., & Jiang, Y. (2013). Cationic core-shell nanoparticles with carmustine contained within O⁶-benzylguanine shell for glioma therapy. *Biomaterials*, *34*(35), 8968–8978. <https://doi.org/10.1016/j.biomaterials.2013.07.097>
85. Beier, C. P., Schmid, C., Gorlia, T., Kleinletzenberger, C., Beier, D., Grauer, O., Steinbrecher, A., Hirschmann, B., Brawanski, A., Dietmaier, C., Jauch-Worley, T., Kölbl, O., Pietsch, T., Proescholdt, M., Rümmele, P., Muigg, A., Stockhammer, G., Hegi, M., Bogdahn, U., & Hau, P. (2009). RNOP-09: pegylated liposomal doxorubicine and prolonged temozolomide in addition to radiotherapy in newly diagnosed glioblastoma--a phase II study. *BMC cancer*, *9*, 308. <https://doi.org/10.1186/1471-2407-9-308>
86. Rodà, F., Caraffi, R., Picciolini, S., Tosi, G., Vandelli, M. A., Ruozi, B., Bedoni, M., Ottonelli, I., & Duskey, J. T. (2023). Recent Advances on Surface-Modified GBM Targeted Nanoparticles: Targeting Strategies and Surface Characterization. *International journal of molecular sciences*, *24*(3), 2496. <https://doi.org/10.3390/ijms24032496>
87. Ying, X., Wen, H., Lu, W. L., Du, J., Guo, J., Tian, W., Men, Y., Zhang, Y., Li, R. J., Yang, T. Y., Shang, D. W., Lou, J. N., Zhang, L. R., & Zhang, Q. (2010). Dual-targeting daunorubicin liposomes improve the therapeutic efficacy of brain glioma in animals. *Journal of controlled release : official journal of the Controlled Release Society*, *141*(2), 183–192. <https://doi.org/10.1016/j.jconrel.2009.09.020>
88. Li, Y., He, H., Jia, X., Lu, W. L., Lou, J., & Wei, Y. (2012). A dual-targeting nanocarrier based on poly(amidoamine) dendrimers conjugated with transferrin and tamoxifen for treating brain gliomas. *Biomaterials*, *33*(15), 3899–3908. <https://doi.org/10.1016/j.biomaterials.2012.02.004>
89. Du, J., Lu, W. L., Ying, X., Liu, Y., Du, P., Tian, W., Men, Y., Guo, J., Zhang, Y., Li, R. J., Zhou, J., Lou, J. N., Wang, J. C., Zhang, X., & Zhang, Q. (2009). Dual-targeting topotecan liposomes modified with tamoxifen and wheat germ agglutinin significantly improve drug transport across the blood-brain barrier and survival of brain tumor-bearing animals. *Molecular pharmaceutics*, *6*(3), 905–917. <https://doi.org/10.1021/mp800218q>
90. Zhang, L., Zhang, Y., Wang, X., Zhou, Y., Qi, J., Gu, L., Zhao, Q., Yu, R., & Zhou, X. (2023). A Trojan-Horse-Like Biomimetic Nano-NK to Elicit an Immunostimulatory Tumor Microenvironment for Enhanced GBM Chemo-Immunotherapy. *Small (Weinheim an der Bergstrasse, Germany)*, *19*(44), e2301439. <https://doi.org/10.1002/smll.202301439>
91. Nelson, D., Fisher, S., & Robinson, B. (2014). The "Trojan Horse" approach to tumor immunotherapy: targeting the tumor microenvironment. *Journal of immunology research*, *2014*, 789069. <https://doi.org/10.1155/2014/789069>
92. Duan, M., Cao, R., Yang, Y., Chen, X., Liu, L., Ren, B., Wang, L., & Goh, B. C. (2024). Blood-Brain Barrier Conquest in Glioblastoma Nanomedicine: Strategies, Clinical Advances, and Emerging Challenges. *Cancers*, *16*(19), 3300. <https://doi.org/10.3390/cancers16193300>
93. Qureshi, S., Anjum, S., Hussain, M., Sheikh, A., Gupta, G., Almoyad, M. A. A., Wahab, S., & Kesharwani, P. (2024). A recent insight of applications of gold nanoparticles in glioblastoma multiforme therapy. *International journal of pharmaceutics*, *660*, 124301. <https://doi.org/10.1016/j.ijpharm.2024.124301>
94. Hao, Y., Zhang, B., Zheng, C., Ji, R., Ren, X., Guo, F., Sun, S., Shi, J., Zhang, H., Zhang, Z., Wang, L., & Zhang, Y. (2015). The tumor-targeting core-shell structured DTX-loaded PLGA@Au nanoparticles for chemo-photothermal therapy and X-ray imaging. *Journal of controlled release : official journal of the Controlled Release Society*, *220*(Pt A), 545–555. <https://doi.org/10.1016/j.jconrel.2015.11.016>
95. Yasri, S., & Wiwanitkit, V. (2017). Important ethical issues for nanomedicine. *Journal of research in medical sciences : the official journal of Isfahan University of Medical Sciences*, *22*, 138. <https://doi.org/10.4103/jrms.JRMS_856_17>
96. Singh, N., Manshian, B., Jenkins, G. J., Griffiths, S. M., Williams, P. M., Maffeis, T. G., Wright, C. J., & Doak, S. H. (2009). NanoGenotoxicology: the DNA damaging potential of engineered nanomaterials. *Biomaterials*, *30*(23-24), 3891–3914. <https://doi.org/10.1016/j.biomaterials.2009.04.009>
97. Bezze, Andrea & Ciardelli, Gianluca & Mattu, Clara. (2023). In vitro human-relevant glioblastoma models as the novel frontier of nanomedicine screening. Biomedical Science and Engineering. 4.  <https://doi.org/10.4081/bse.222>
98. Schulz, J. A., Rodgers, L. T., Kryscio, R. J., Hartz, A. M. S., & Bauer, B. (2022). Characterization and comparison of human glioblastoma models. *BMC cancer*, *22*(1), 844. <https://doi.org/10.1186/s12885-022-09910-9>
99. Tatla, A. S., Justin, A. W., Watts, C., & Markaki, A. E. (2021). A vascularized tumoroid model for human glioblastoma angiogenesis. *Scientific reports*, *11*(1), 19550. <https://doi.org/10.1038/s41598-021-98911-y>
100. Gómez-Oliva, R., Domínguez-García, S., Carrascal, L., Abalos-Martínez, J., Pardillo-Díaz, R., Verástegui, C., Castro, C., Nunez-Abades, P., & Geribaldi-Doldán, N. (2021). Evolution of Experimental Models in the Study of Glioblastoma: Toward Finding Efficient Treatments. *Frontiers in oncology*, *10*, 614295. <https://doi.org/10.3389/fonc.2020.614295>
101. Zhao, C., Zhu, X., Tan, J., Mei, C., Cai, X., & Kong, F. (2024). Lipid-based nanoparticles to address the limitations of GBM therapy by overcoming the blood-brain barrier, targeting glioblastoma stem cells, and counteracting the immunosuppressive tumor microenvironment. *Biomedicine & pharmacotherapy = Biomedecine & pharmacotherapie*, *171*, 116113. <https://doi.org/10.1016/j.biopha.2023.116113>
102. Stanković, T., Ranđelović, T., Dragoj, M., Stojković Burić, S., Fernández, L., Ochoa, I., Pérez-García, V. M., & Pešić, M. (2021). In vitro biomimetic models for glioblastoma-a promising tool for drug response studies. *Drug resistance updates : reviews and commentaries in antimicrobial and anticancer chemotherapy*, *55*, 100753. <https://doi.org/10.1016/j.drup.2021.100753>
103. Malik, S., Muhammad, K., & Waheed, Y. (2023). Nanotechnology: A Revolution in Modern Industry. *Molecules (Basel, Switzerland)*, *28*(2), 661. <https://doi.org/10.3390/molecules28020661>
104. Ding, W., Zhou, X., Jiang, G., Xu, W., Long, S., Xiao, F., Liao, Y., & Liu, J. (2022). Identification of Prognostic Biomarkers of Glioblastoma Based on Multidatabase Integration and Its Correlation with Immune-Infiltration Cells. *Journal of oncology*, *2022*, 3909030. <https://doi.org/10.1155/2022/3909030>
105. Erickson, A., Jackson, L. R., Camphausen, K., & Krauze, A. V. (2024). Mucins as Precision Biomarkers in Glioma: Emerging Evidence for Their Potential in Biospecimen Analysis and Outcome Prediction. *Biomedicines*, *12*(12), 2806. <https://doi.org/10.3390/biomedicines12122806>
106. Kaushik Ajeet. (2019). Biomedical Nanotechnology Related Grand Challenges and Perspectives. *Frontiers in Nanotechnology.* Volume 1.  <https://doi.org/10.3389/fnano.2019.00001>
107. Hu, D., Xia, M., Wu, L., Liu, H., Chen, Z., Xu, H., He, C., Wen, J., & Xu, X. (2023). Challenges and advances for glioma therapy based on inorganic nanoparticles. *Materials today. Bio*, *20*, 100673. <https://doi.org/10.1016/j.mtbio.2023.100673>
108. Sharma, Krati. (2015). Book - Nanobiotechnology for sensing applications: From lab to field.
109. Gomes, M., Ramalho, M. J., Loureiro, J. A., & Pereira, M. C. (2025). Advancing Brain Targeting: Cost-Effective Surface-Modified Nanoparticles for Faster Market Entry. *Pharmaceutics*, *17*(5), 661. <https://doi.org/10.3390/pharmaceutics17050661>
110. Madani, F., Morovvati, H., Webster, T. J., Najaf Asaadi, S., Rezayat, S. M., Hadjighassem, M., Khosravani, M., & Adabi, M. (2024). Combination chemotherapy via poloxamer 188 surface-modified PLGA nanoparticles that traverse the blood-brain-barrier in a glioblastoma model. *Scientific reports*, *14*(1), 19516. <https://doi.org/10.1038/s41598-024-69888-1>
111. Noorani, I., de la Rosa, J. (2023). Breaking barriers for glioblastoma with a path to enhanced drug delivery. *Nat Commun* 14, 5909. <https://doi.org/10.1038/s41467-023-41694-9>
112. Chan, M. H., Chen, W., Li, C. H., Fang, C. Y., Chang, Y. C., Wei, D. H., Liu, R. S., & Hsiao, M. (2021). An Advanced *In Situ* Magnetic Resonance Imaging and Ultrasonic Theranostics Nanocomposite Platform: Crossing the Blood-Brain Barrier and Improving the Suppression of Glioblastoma Using Iron-Platinum Nanoparticles in Nanobubbles. *ACS applied materials & interfaces*, *13*(23), 26759–26769. <https://doi.org/10.1021/acsami.1c04990>
113. Zhao, X., Zhao, H., Chen, Z., & Lan, M. (2014). Ultrasmall superparamagnetic iron oxide nanoparticles for magnetic resonance imaging contrast agent. *Journal of nanoscience and nanotechnology*, *14*(1), 210–220. <https://doi.org/10.1166/jnn.2014.9192>
114. Xiaoxuan Zhong, Xiang Wei, Yan Xu, Xuehai Zhu, Bo Huo, Xian Guo, Gaoke Feng, Zihao Zhang, Xin Feng, Zemin Fang, Yuxuan Luo, Xin Yi, Ding-Sheng Jiang- (2024). The lysine methyltransferase SMYD2 facilitates neointimal hyperplasia by regulating the HDAC3–SRF axis. *Acta Pharmaceutica Sinica B, Volume 14, Issue 2.* <https://doi.org/10.1016/j.apsb.2023.11.012>.
115. Kuo, Y. C., Lin, P. I., & Wang, C. C. (2011). Targeting nevirapine delivery across human brain microvascular endothelial cells using transferrin-grafted poly(lactide-co-glycolide) nanoparticles. *Nanomedicine (London, England)*, *6*(6), 1011–1026. <https://doi.org/10.2217/nnm.11.25>
116. Maggiorella, L., Barouch, G., Devaux, C., Pottier, A., Deutsch, E., Bourhis, J., Borghi, E., & Levy, L. (2012). Nanoscale radiotherapy with hafnium oxide nanoparticles. *Future oncology (London, England)*, *8*(9), 1167–1181. <https://doi.org/10.2217/fon.12.96>
117. Johannsen, M., Thiesen, B., Jordan, A., Taymoorian, K., Gneveckow, U., Waldöfner, N., Scholz, R., Koch, M., Lein, M., Jung, K., & Loening, S. A. (2005). Magnetic fluid hyperthermia (MFH)reduces prostate cancer growth in the orthotopic Dunning R3327 rat model. *The Prostate*, *64*(3), 283–292. <https://doi.org/10.1002/pros.20213>
118. Lucky, S. S., Soo, K. C., & Zhang, Y. (2015). Nanoparticles in photodynamic therapy. *Chemical reviews*, *115*(4), 1990–2042. <https://doi.org/10.1021/cr5004198>
119. Riley, R. S., & Day, E. S. (2017). Gold nanoparticle-mediated photothermal therapy: applications and opportunities for multimodal cancer treatment. *Wiley interdisciplinary reviews. Nanomedicine and nanobiotechnology*, *9*(4), 10.1002/wnan.1449. <https://doi.org/10.1002/wnan.1449>
120. Camille Verry, Sandrine Dufort, Julie Villa, Marylaure Gavard, Carole Iriart, Sylvie Grand, Julie Charles, Benoit Chovelon, Jean-Luc Cracowski, Jean-Louis Quesada, Christophe Mendoza, Lucie Sancey, Audrey Lehmann, Florence Jover, Jean-Yves Giraud, François Lux, Yannick Crémillieux, Stephen McMahon, Petrus J. Pauwels, Daniel Cagney, Ross Berbeco, Ayal Aizer, Eric Deutsch, Markus Loeffler, Géraldine Le Duc, Olivier Tillement, Jacques Balosso. (2021). Theranostic AGuIX nanoparticles as radiosensitizer: A phase I, dose-escalation study in patients with multiple brain metastases (NANO-RAD trial). *Radiotherapy and Oncology, Volume 160.* <https://doi.org/10.1016/j.radonc.2021.04.021>.
121. Kunjachan, S., Ehling, J., Storm, G., Kiessling, F., & Lammers, T. (2015). Noninvasive Imaging of Nanomedicines and Nanotheranostics: Principles, Progress, and Prospects. *Chemical reviews*, *115*(19), 10907–10937. <https://doi.org/10.1021/cr500314d>
122. Patil R, Gangalum PR, Wagner S, et al. A nanoparticle-based approach to selectively block the angiogenic effects of VEGF165 in glioblastoma. *Neuro Oncol*. 2015;17(3):392–402. PMID: 25228635. <https://doi.org/10.1093/neuonc/nou193>
