# Supplementary material for: Advancing glioblastoma therapy with surface-modified nanoparticles
Source: Neurol Sci. 2025 Sep 13;46(11):5757–70. doi: 10.1007/s10072-025-08457-4 (PMC12537612; doi:10.1007/s10072-025-08457-4)
Supplement: Supplementary file 2 — corrected proofs with references and citations in main tex: unable to load [file 10072_2025_8457_MOESM2_ESM.pdf]

Metadata of the article that will be visualized online

ArticleTitle		Advancing glioblastoma therapy with surface-modified nanoparticles
Article CopyRight - Year		Fondazione Società Italiana di Neurologia 2025

Corresponding Author	Family Name	Ius
	Particle	
	Given Name	Tamara
	Organization	University of Padova
	Address	Padova 35121, Italy
	Division	Academic Neurosurgery, Department of Neurosciences
	Email	tamara.ius@gmail.com
Author	Family Name	Rosa
	Particle	De
	Given Name	Giorgia
	Organization	University of Brescia
	Address	Piazza Spedali Civili 1 Brescia 25123, Italy
	Division	Division of Neurosurgery, Department of Medical and Surgical Specialties, Radiological Sciences and Public Health
	Email	g.derosa003@studenti.unibs.it
Author	Family Name	Zeppieri
	Particle	
	Given Name	Marco
	Organization	University Hospital of Udine
	Address	p.le S. Maria della Misericordia 15 Udine 33100, Italy
	Division	Department of Ophthalmology
	Email	markzeppieri@hotmail.com
Author	Family Name	Zeppieri
	Particle	
	Given Name	Marco
	Organization	University of Trieste
	Address	Trieste 34127, Italy
	Division	Department of Medicine, Surgery and Health Sciences
	Email	markzeppieri@hotmail.com
Author	Family Name	Gagliano
	Particle	

	Given Name	Caterina
	Organization	University of Enna″Kore″
	Address	Piazza dell'Università Enna 94100, Italy
	Division	Department of Medicine and Surgery
	Email	caterina.gagliano@unikore.it
Author	Family Name	Gagliano
	Particle	
	Given Name	Caterina
	Organization	Mediterranean Foundation″G.B. Morgagni″
	Address	Catania 95125, Italy
	Email	caterina.gagliano@unikore.it
Author	Family Name	Tel
	Particle	
	Given Name	Alessandro
	Organization	University Hospital of Udine
	Address	Udine, Italy
	Division	Clinic of Maxillofacial Surgery, Head-Neck and NeuroScience Department
	Email	alessandro.tel@icloud.com
Author	Family Name	Tognetto
	Particle	
	Given Name	Daniele
	Organization	University of Trieste
	Address	Trieste 34127, Italy
	Division	Department of Medicine, Surgery and Health Sciences
	Email	tognetto@units.it
Author	Family Name	Panciani
	Particle	
	Given Name	Pier Paolo
	Organization	University of Brescia
	Address	Piazza Spedali Civili 1 Brescia 25123, Italy
	Division	Division of Neurosurgery, Department of Medical and Surgical Specialties, Radiological Sciences and Public Health
	Email	pierpaolo.panciani@unibs.it
Author	Family Name	Fontanella
	Particle	
	Given Name	Marco Maria
	Organization	University of Brescia

	Address	Piazza Spedali Civili 1 Brescia 25123, Italy
	Division	Division of Neurosurgery, Department of Medical and Surgical Specialties, Radiological Sciences and Public Health
	Email	marco.fontanella@unibs.it
Author	Family Name	Agosti
	Particle	
	Given Name	Edoardo
	Organization	University of Brescia
	Address	Piazza Spedali Civili 1 Brescia 25123, Italy
	Division	Division of Neurosurgery, Department of Medical and Surgical Specialties, Radiological Sciences and Public Health
	Email	edoardo_agosti@libero.it
Schedule	Received	19 April 2025
	Revised	
	Accepted	16 August 2025

Abstract

Background

Glioblastoma multiforme (GBM) is a very aggressive and deadly brain tumor, presenting considerable therapeutic hurdles due to its infiltrative development, heterogeneity, and protective mechanisms of the blood-brain barrier (BBB). Traditional treatment methods frequently do not yield satisfactory results, requiring the implementation of novel solutions. Surface-modified nanoparticles (NPs) have emerged as a viable approach in GBM therapy, providing potential benefits in targeted drug delivery, improved therapeutic efficacy, and reduced systemic toxicity. Aim: This narrative review examines progress in the creation and utilization of surface-modified NPs, emphasizing their function in traversing the blood-brain barrier and selectively targeting glioblastoma cells.

Methods

This review consolidates findings from an extensive search of principal medical databases, highlighting in vitro, in vivo, and ex vivo investigations on surface-modified NPs in the treatment of GBM. The discourse emphasizes diverse methodologies, surface alteration procedures, and their ramifications for therapeutic effectiveness and clinical relevance.

Results

In the last ten years, considerable advancements have been achieved in customizing NPs for targeting GBM. Surface modifications, including conjugation with ligands, peptides, or polymers, have significantly enhanced NP stability, biocompatibility, and specificity. Receptor-mediated targeting has been a primary method, utilizing unique molecular markers that are overexpressed on GBM cells to improve the precision of drug delivery. Dual-targeting strategies that focus on both the blood-brain barrier and tumor microenvironment have demonstrated promise in enhancing therapeutic results. Moreover, sophisticated surface characterization methods have yielded essential insights on NP efficacy, guaranteeing the dependability and consistency of these systems. Preclinical models, especially in vivo studies, have highlighted the translational potential of these methods, showing enhanced medication penetration and efficacy in difficult GBM scenarios.

Conclusions

Surface-modified NPs signify a groundbreaking advancement in GBM therapy, providing novel answers to persistent difficulties. By combining innovative surface engineering with tailored therapeutic administration, they aim to improve treatment accuracy and reduce off-target consequences. Nevertheless, substantial obstacles persist, such as tackling NP toxicity, enhancing surface modification techniques, and guaranteeing scalability for clinical use.

Keywords(seperated by –)

Surface modified nanoparticles–
Glioblastoma–
Brain-blood barrier–
Tumor microenvironment–

Neurological Sciences
https://doi.org/10.1007/s10072-025-08457-4

REVIEW ARTICLE

Advancing glioblastoma therapy with surface-modified nanoparticles

Giorgia De Rosa[1] · Marco Zeppieri[2,3] · Caterina Gagliano[4,5] · Alessandro Tel[6] · Daniele Tognetto[3] · Pier Paolo Panciani[1] · Marco Maria Fontanella[1] · Tamara Ius[7] · Edoardo Agosti[1]

Received: 19 April 2025 / Accepted: 16 August 2025
© Fondazione Società Italiana di Neurologia 2025

UNCORRECTED PROOF

Abstract

Background Glioblastoma multiforme (GBM) is a very aggressive and deadly brain tumor, presenting considerable therapeutic hurdles due to its infiltrative development, heterogeneity, and protective mechanisms of the blood-brain barrier (BBB). Traditional treatment methods frequently do not yield satisfactory results, requiring the implementation of novel solutions. Surface-modified nanoparticles (NPs) have emerged as a viable approach in GBM therapy, providing potential benefits in targeted drug delivery, improved therapeutic efficacy, and reduced systemic toxicity. Aim: This narrative review examines progress in the creation and utilization of surface-modified NPs, emphasizing their function in traversing the blood-brain barrier and selectively targeting glioblastoma cells.

AQ1

Methods This review consolidates findings from an extensive search of principal medical databases, highlighting in vitro, in vivo, and ex vivo investigations on surface-modified NPs in the treatment of GBM. The discourse emphasizes diverse methodologies, surface alteration procedures, and their ramifications for therapeutic effectiveness and clinical relevance.

AQ2

Results In the last ten years, considerable advancements have been achieved in customizing NPs for targeting GBM. Surface modifications, including conjugation with ligands, peptides, or polymers, have significantly enhanced NP stability, biocompatibility, and specificity. Receptor-mediated targeting has been a primary method, utilizing unique molecular markers that are overexpressed on GBM cells to improve the precision of drug delivery. Dual-targeting strategies that focus on both the blood-brain barrier and tumor microenvironment have demonstrated promise in enhancing therapeutic results. Moreover, sophisticated surface characterization methods have yielded essential insights on NP efficacy, guaranteeing the dependability and consistency of these systems. Preclinical models, especially in vivo studies, have highlighted the translational potential of these methods, showing enhanced medication penetration and efficacy in difficult GBM scenarios.

Conclusions Surface-modified NPs signify a groundbreaking advancement in GBM therapy, providing novel answers to persistent difficulties. By combining innovative surface engineering with tailored therapeutic administration, they aim to improve treatment accuracy and reduce off-target consequences. Nevertheless, substantial obstacles persist, such as tackling NP toxicity, enhancing surface modification techniques, and guaranteeing scalability for clinical use.

Keywords Surface modified nanoparticles · Glioblastoma · Brain-blood barrier · Tumor microenvironment

Introduction

AQ3

Glioblastoma multiforme (GBM) represents 45.6% of primary malignant brain tumors, with an annual incidence of 3.1 per 100 000 [1] and a median survival of <15 months [2–4]. According to the latest World Health Organization (WHO) Classification of Tumors of the Central Nervous System (CNS), published in 2021 [5, 6], all GBMs are now by definition IDH-wildtype, making them the most aggressive form of brain tumors. This, along with difficult drug delivery through the blood-brain barrier (BBB) and with an immunosuppressive tumor microenvironment (TME),

Co-first: Giorgia De Rosa and Marco Zeppieri; the authors participated equally.

Co-last: Tamara Ius and Edoardo Agosti; the authors participated equally.

Extended author information available on the last page of the article

explains why current chemotherapies do not yield satisfactory results, requiring novel solutions [7, 8]. New therapeutical strategies, such as immunotherapy, gene therapy, oncolytic virotherapy, stem cell therapy, photodynamic therapy, and hyperthermia therapy [9], are being developed but none of these will have an impact until a way to bypass the BBB and the TME will be found. In this paper, we will explore the challenge of drug transport across the BBB. We will start with a brief overview of this physical and functional barrier to understand how specific receptors on the surface of vascular endothelial cells can be leveraged for their ability to bind ligands, enabling targeted therapy, and facilitating the passage of various substances through the BBB. Among these, surface-modified nanoparticles (NPs) have gained increasing attention over the past decade. Additionally, we will examine the tumor microenvironment (TME) and its intricate crosstalk with GBM stem cells (GSCs), explaining how these emerging drug delivery systems (DDSs) are designed to target tumor cells and disrupt tumor proliferation. Through this narrative review, we aim to provide a comprehensive analysis of the existing literature on these novel DDSs, highlighting their benefits and risks while acknowledging that the lack of a universal marker for effective targeting remains a significant challenge in this field of research.

Scope clarification

Given the multidisciplinary and rapidly evolving nature of nanoparticle-based therapies for GBM, this review was intentionally focused on surface-functionalized nanoparticles, with particular emphasis on their role in blood–brain barrier traversal and targeted delivery to tumor tissue. Several complementary topics—such as theranostic strategies (e.g., LITT, FUS), magnetic hyperthermia, photothermal and photodynamic therapy, radiotherapy-conjugated platforms, patient-derived organoid models, and AI-driven nanoparticle design—are highly relevant to the broader translational landscape, they are beyond the specified scope of this manuscript. We intentionally decided to exclude all these aspects in order to keep the review focused and to provide a deeper discussion of surface modification strategies. However, we recognize the importance of these emerging areas and believe they should be addressed in future dedicated reviews.

Materials and methods

This narrative review aims to provide a comprehensive synthesis of recent advancements in the use of surface-modified NPs for the treatment of GBM. Given the interdisciplinary nature of this field, the methodology was designed to integrate a broad range of high-quality, relevant sources while maintaining scientific rigor. A systematic literature search was conducted to identify peer-reviewed articles published up to December 2024. The databases searched included PubMed, Scopus, and Ovid MEDLINE. Search terms were structured around the following keyword combinations: "surface-modified nanoparticles" AND "glioblastoma," "nanocarriers" AND "blood-brain barrier," and "tumor microenvironment" AND "targeted drug delivery." Boolean operators (AND, OR) and Medical Subject Headings (MeSH) were applied as appropriate to refine the search.

Inclusion criteria were as follows: (i) original articles published in English; (ii) studies involving surface-functionalized or surface-modified NPs targeting GBM; and (iii) in vitro or in vivo experimental studies, particularly those addressing BBB penetration, tumor targeting, or modulation of the tumor microenvironment. Exclusion criteria included: (i) non-English publications; (ii) studies that did not explicitly investigate NP surface modifications; and (iii) conference abstracts, editorials, and papers lacking primary data.

Two independent authors screened titles and abstracts to assess relevance to the central topic of NP-based GBM targeting. Full texts of potentially eligible studies were then reviewed in detail. Any disagreements were resolved by consensus. The review focused on research elucidating core principles of NP-cell interactions, ligand-receptor targeting strategies, and their translational potential in GBM models. Findings were thematically summarized across key areas: strategies for physicochemical modification, blood-brain barrier traversal, intratumoral distribution, and therapeutic outcomes.

Results

The comprehensive literature search conducted for this review yielded 91 peer-reviewed articles that met the established inclusion criteria. These studies were selected based on their relevance to the role of surface-modified NPs in the treatment of GBM, with particular focus on BBB penetration, tumor specificity, modulation of the TME, and therapeutic efficacy. The publications spanned from 2005 to December 2024, with the majority appearing within the past five years, reflecting growing interest and rapid progress in this interdisciplinary field. Among the selected studies, 37 were exclusively in vitro, 35 utilized in vivo animal models,

UNCORRECTED PROOF

and 19 employed both experimental approaches. Although most investigations remain at the preclinical stage, they collectively provide substantial insight into the design, performance, and translational promise of surface-engineered NP platforms for GBM therapy.

A central theme across the literature is the critical importance of physicochemical surface engineering in enabling NPs to traverse the BBB. Unmodified NPs are often rapidly cleared and exhibit poor BBB permeability. In contrast, PEGylation has been widely employed to extend systemic circulation and reduce immune recognition. Concurrently, ligand-based functionalization - using agents such as transferrin, lactoferrin, angiopep-2, and folic acid - facilitates receptor-mediated transcytosis across the BBB, enhancing brain accumulation by 2- to 5-fold in preclinical models. Once across the BBB, tumor specificity is achieved through ligands targeting overexpressed receptors such as EGFRvIII, IL13Rα2, and CD44.

Hyaluronic acid-modified NPs exhibit strong affinity for CD44-overexpressing GSCs, thereby reducing therapeutic resistance and recurrence. Similarly, aptamer-mediated targeting of nucleolin and PDGFRβ has demonstrated comparable selectivity. Additional innovations include NPs incorporating MMP-cleavable linkers or pH-responsive coatings that undergo structural transformation within the TME, enhancing intratumoral penetration and distribution. Mannose-functionalized NPs have also shown immunomodulatory potential by reprogramming tumor-associated macrophages (TAMs) from the immunosuppressive M2 phenotype to the pro-inflammatory M1 phenotype, resulting in increased T cell infiltration and tumor suppression. These mechanistically driven surface modifications have led to notable therapeutic outcomes in preclinical evaluations.

Both monotherapy NPs and dual-delivery systems, such as temozolomide co-delivered with MGMT-targeting siRNA, have consistently outperformed conventional therapies, significantly reducing tumor burden and extending survival in animal models. Median survival in treated cohorts ranged from 38 to 57 days, compared to 22 to 31 days in control groups. Advanced imaging modalities confirmed targeted accumulation within tumors, with tumor-to-brain uptake ratios frequently exceeding 5:1.

Safety profiles across the studies were largely favorable. PEGylated and polysorbate-coated NPs showed no signs of systemic toxicity, and most hematologic and hepatic parameters remained within normal limits. Nonetheless, concerns persist regarding the potential for chronic immunogenicity following repeated administration of ligand-functionalized platforms. Overall, the compiled evidence underscores the pivotal role of surface modification in enhancing the in vivo performance of NPs, governing their pharmacokinetics, biodistribution, tumor targeting, and intracellular delivery.

Despite encouraging preclinical outcomes, the field remains at a critical translational inflection point. Standardization of NP formulations, validation in clinically relevant models, and resolution of regulatory hurdles will be essential for advancing these technologies from the laboratory into clinical practice. The following sections of this review examine these challenges in greater detail.

Discussion

GBM is a highly heterogeneous tumor, characterized by intricate spatial and temporal heterogeneity that substantially contributes to therapy failure and early recurrence [10, 11]. Despite maximal safe resection and standard chemoradiotherapy, most recurrences arise within 2 cm margin of the first surgery, indicating the persistence of infiltrative tumor cells and the inadequacies of systemic treatments [12]. These challenges have prompted the creation of innovative locoregional treatment techniques designed to improve drug delivery in the peritumoral area and leverage the distinctive biological characteristics of the tumor microenvironment. In this context, surface-modified nanoparticles present a promising approach, providing targeted delivery capabilities and potential integration with existing local therapies to more effectively address residual disease.

Blood-Brain barrier and drug delivery mechanisms

Blood-brain barrier is composed of endothelial cells of the capillary wall, astrocyte end-feet, and pericytes [13]. More specifically, it is the result of tight junctions between the endothelial cells of brain capillaries, whose role is to regulate the movement of molecules, ions, and cells between the blood and the CNS [14]. The protective nature of this barrier provides both a defense mechanism from pathogens and toxic substances, and an obstacle for drugs delivery. The structure is further supported by the basal lamina, thick matrix mainly formed by collagen type IV, heparin sulphate proteoglycans, fibronectin, laminin. Multiple basal lamina proteins, matrix metalloproteases (MMPs) and their inhibitors are responsible for the integrity of the BBB and are exploited, due to their dramatically upregulated expression in GBMs, to enhance targeted therapies. Other molecules hyper-expressed in the BBB are transferrin (Tf), insulin, low-density lipoprotein, lactoferrin (Lf), nicotinic receptors, and glucose and choline transporters [15–21]. They have all been studied for GBM treatment, but recent studies proved that targeting a single molecule has limited efficacy. An ideal solution would be to select multiple ligands to increase the chances of crossing the BBB and many studies are focusing,

UNCORRECTED PROOF

indeed, on multi-ligand functionalized nanomedicine and on dual targeting both the BBB and the tumor cells [12, 22].

Although acting as a selective barrier, the BBB does allow substances to enter, both through gradient-driven or energy-dependent transport. The former represents a passive transport, and it is exactly what current therapeutics take advantage of. However, most of the drugs efficient against GBM do not possess the characteristics to exploit this easy route to the brain, hence the need to exploit different mechanisms, such as para-cellular transport, carrier-mediated transport, or receptor-mediated transcytosis.

Tumors, in particular high-grade gliomas, exploit the BBB not only passively, taking advantage of its role as a semi-permeable barrier that creates the so-called "pharmacological sanctuary", but also actively, through tumor-mediated changes: because of their increased metabolism, leading to local hypoxia, hypoxia-inducible-factor (HIF) is overproduced and stimulates vascular endothelial growth factor (VEGF). VEGF alters the BBB architecture and induces the formation of abnormal capillaries. This tumor-regulated neoangiogenesis concurs to accommodate the high metabolic demands of glioma cells, guaranteeing their survival and constant proliferation [23, 24].

Tumor microenvironment and glioblastoma stem cells

TME is the region surrounding the primary lesion. It acts as a dynamic and active component of the tumor itself, due to its immunosuppressive nature and its ability to favor metastatic diffusion. The simple existence of these two characteristics led to think that there must be a strong connection between the tumor and its surrounding, connection that was found to lie in the crosstalk between the TME and GSCs. The latter produce neutrosphere-like cell clusters with high CD133 expression that function as trophic agents to the TME, with self-renewal and tumorigenic capacity [25–28]. Among all the GSCs markers, CD133 is one of the most studied due to its association with low survival rates since it can influence recurrence, prognosis and aggressiveness [29–31]. However, GBMs CD133 negative have been identified, proving the heterogeneity that distinguishes this pathology and confirming the lack of a single marker towards which all efforts can be directed. Integrin-α6 and CD44 are other markers often co-expressed with CD133, so most of the studies revolve around them.

Alternatively, another solution is to target the TME instead of GSCs receptors, either by targeting endothelial cells or pathways involved with angiogenesis i.e. VEGFR [32, 33]. Targeting the tumor by exploiting its TME characteristics was a central theme in the 90 s when the enhanced permeation and retention effect (EPR) was first observed. It was basically because of the EPR effect that nanomedicine started to develop to guarantee accumulation at tumor sites [34]. However, recent analysis proved that the EPR effect is more present in rodents than in humans and that it has a considerable heterogeneity among different patients and among different tumors, meaning that it alone cannot guarantee the accumulation of NPs in tumoral cells. Because of this conclusion, NPs have been further modified [35–38]. For example, the addition of collagenase or hyaluronic acid can respectively promote extravasation, following interaction with the CD44 receptor, and improve penetration and accumulation at the tumor site [39].

The epidermal growth factor (EGF) and VEGF, whose role has already been discussed in this paper, represent a target of research because of their hyper-expression in the tumor site. However, the main problem with VEGF, is that anti-angiogenic treatments are often useless in highly innervated tumors. VEGF-targeted NPs could stimulate a response from tumor-associated nerves (TANs) that would annihilate the therapy by restoring angiogenesis [40–42].

Moreover, one of the most predominant cell populations in TME is the one of tumor associated macrophages (TAMs) that have a role in neovascularization, hence in tumor proliferation. These cells are recruited by GSC-induced periostin secretion, meaning that silencing periostin can be considered another valid alternative to target the TME and alter the tumor proliferation.

Surface-Modified nanoparticles: design and targeting strategies

NPs are nano-sized molecules whose small sizes, low toxicity and controlled drug release profile [43] won them a key role in recent studies about target therapies against GBM. Additionally, their surface can be modified with targeting ligands, allowing them to localize drug delivery through the blood-brain barrier and into gliomas. Their application in the medical field, known as nanomedicine, allow them to carry traditional drugs to enable passage through the BBB and to GBM cells via targeting, control release at the target site and reduce off-target toxicity [44–49].

They are classified as organic, inorganic and biological carriers with liposomes and polymeric NPs being the most successful among the organic ones and carbon nanotubes (CNTs), and gold NPs (Au-NPs) leading the inorganic category. Given the hyper selectivity of the BBB, however, biomimetic devices such as NPs coated with erythrocyte membranes, showed a better efficacy in overcoming this obstacle [50–52].

We have already discussed active and passive transport through the BBB. Theoretically, NPs could exploit both these mechanisms, however anti-tumor drugs are usually

UNCORRECTED PROOF

larger molecules, meaning that diffusion-dependent routes are inapplicable. Thus, energy-dependent routes are the delivery mechanism of choice [53–55].

Regarding the dimensions, there is no universal optimal size. Most studies on drug delivery through the BBB use NPs in size from 10 to 100 nm, but factors such as the type of NP, associated surface proteins and physiological functioning of the BBB should be considered [56].

The protein corona (PC) is another fundamental aspect of NPs, functional to its efficiency. It is defined as “the outer layer of deliverables” and it is on its interaction with both the NP and the surrounding environment that drugs delivery depends. The thicker and the stabler the PC, the better the outcome. These two characteristics are correlated to the size and concentration of the NPs [57, 58].

The most investigated surface modification is the one of Poly ethylene glycol-poly lactic acid (PEG-PLA) NPs, due to their long circulating behavior in the blood stream after intravenous or intranasal administration [59]. Besides, PEG and PLA are both materials approved by the Food and Drug Administration, ensuring safety to the studies. However, PEG chains inhibit interaction with cell surfaces, hindering BBB penetration. A solution was found in conjugating it to an activatable low molecular weight protamine (ALMWP) to form a cell penetrating peptides able to enhance the targeted therapy and to penetrate the tumor. Because of all these characteristics, ALMWP-NP was then loaded with anti-tumor agents effective on GBM, for example Paclitaxel (PTX) [60]. PTX interferes with the normal breakdown of microtubules, preventing cell division and leading to cell death [61]. Currently approved formulations of PTX lack the ability to pass through the blood-brain barrier, hence the need to associate it to ALMW-NP.

Another well studied molecule added to NPs surface is transferrin, commonly used to transport iron across the blood-brain barrier. Transferrin receptors are restricted to brain capillaries, rendering them a potential for targeting therapy through their internalization of Tf via receptor-mediated endocytosis [56, 62, 63]. However, a high concentration of endogenous Tf saturate the receptors, making this system far from being ideal. Thus, antibodies with affinity for different epitopes on the Tf receptor are being investigated i.e. OX26 anti-Tf receptor monoclonal antibody, though current in vivo experiments showed that they are not able to mediate actual crossing of the endothelial cell layer, meaning that more studies will be required [64–66].

Melanotransferrin (p97) is a GPI-anchored protein expressed in melanomas very similar to Tf that instead of Tf receptor, are transported through the endothelium by low-density lipoprotein-receptor related protein (LRP). Not only this has proven to be an efficient DDS, but, in contrast with Tf, plasma concentration of endogenous p97 is relatively low, hence does not saturate binding sites [67, 68]. Accordingly, relative to the Tf receptor system, the melanotransferrin-receptor system emerges as a preferred targeting vector for drug transport into the brain.

Other molecules can be used to modify NPs surfaces, including proteins and antibodies targeting the insulin receptor, the low-density lipoprotein receptor and the leptin receptor. However, all these still present challenges related to collateral effect for glucose metabolism, lack of brain capillary endothelium specificity and, regarding the leptin system, an inappropriate indication in obese individuals.

Small molecules were assessed too, nucleoside adenosine for example was considered due to its involvement in neuronal and synaptic function. Adenosine proved to be able to reduce tight junction cohesion and, accordingly, to increase the BBB permeability. However, it also showed an inefficacy in crossing the BBB due to its short circulation time [69–72]. Hence an association of adenosine to squalene NPs to protect it from metabolization was experimented, resulting in an increase of its circulation time [73].

One more promising surface modification is represented by RGD (Arg-Gly-Asp) peptide sequence, a cell adhesion motif found in many extracellular matrix proteins. Its role in cell attachment, migration, and differentiation turned a spotlight on this molecule, leading to interesting results. RGD peptides bind integrin receptors, inhibit tumor migration and angiogenesis, and can also target the delivery of anti-tumor drugs. Recent studies associated RGD peptides with silver NPs (Ag NPs) and nano-selenium (Se NPs). This DDS induced glioma cells ROS production, decreased mitochondrial membrane potential, and caused MAPKs activation, ultimately resulting in tumor cell apoptosis [74, 75].

A comparative summary of the main surface modification strategies, their mechanisms of action, advantages, and limitations is provided in Table 1.

In parallel with conventional chemical and biological functionalization, recent investigations have explored the potential of green-synthesized nanoparticles in oncology, offering a sustainable and biocompatible alternative for targeted therapies. Green-synthesized nanoparticles have lately attracted interest in nanomedicine because of their excellent biocompatibility, affordability, and potential therapeutic and diagnostic uses. These eco-friendly nanocarriers are being progressively investigated for targeted cancer treatments. Montazersaheb et al. shown that silver nanoparticles derived from pumpkin peel serve as efficient radiosensitizers in triple-negative breast cancer [76]. Rosic et al. examined the function of nanobiomaterials in cancer signaling and gene therapy, highlighting its diagnostic and therapeutic applications [77]. Güneş et al. synthesized silver-coated iron oxide nanoparticles utilizing Hibiscus esculentus, exhibiting antibacterial and magnetic characteristics

UNCORRECTED PROOF

Table 1 Summary of Surface-Modified nanoparticle strategies for glioblastoma treatment this table provides a concise overview of key nanoparticle (NP) strategies explored for glioblastoma multiforme (GBM), focusing on their targeting mechanisms, therapeutic benefits, and limitations in clinical translation

Targeting Strategy	Mechanism of Action	Advantages	Limitations
Passive Targeting (EPR effect)	Exploits leaky tumor vasculature to accumulate nanoparticles passively.	Simple design; effective in rodent models.	Limited efficacy in human GBM due to less permeable vasculature.
Ligand-Mediated Targeting	Targets overexpressed receptors on BBB or GBM cells (e.g., transferrin, CD44).	High specificity; reduced off-target effects.	Requires precise receptor characterization; may vary between patients.
Dual-Ligand Functionalization	Combines multiple receptor-targeting ligands for enhanced selectivity.	Enhanced BBB crossing and tumor selectivity.	Complex synthesis and stability concerns.
Stimuli-Responsive Systems	Alters size/charge in response to pH, enzymes, or redox conditions in the tumor.	Improved penetration and release in TME.	Requires accurate tumor microenvironment mapping.
Immunomodulatory NPs	Reprograms tumor-associated macrophages or delivers immune agents to reshape the TME.	Enhances immune response; synergistic with checkpoint inhibitors.	Potential off-target immune activation; requires safety validation.

[78]. Lastly, Keskin et al. (2025) validated the cytotoxic and antibacterial properties of green silver nanoparticles derived from Anchusa officinalis [79].

Surface characterization techniques

As previously discussed, the absence of a universal marker for effective targeting remains a major obstacle in the advancement of NP-based therapies. Compounding this issue is the widespread problem of incomplete nanomaterial characterization, which continues to hinder progress in the field. Although this limitation is well recognized within the scientific community, it remains challenging to address due to several intrinsic factors. Inadequate characterization not only impedes a comprehensive understanding of nanomaterials, but also undermines the reproducibility of experimental results, which is a critical barrier to translating promising in vitro findings into clinical applications [80].

Nonetheless, several surface characterization techniques are currently available. Among those most frequently employed in the literature we reviewed are zeta potential measurement and X-ray photoelectron spectroscopy. Zeta potential analysis evaluates the electrostatic repulsion between similarly charged particles, providing insight into colloidal stability and surface charge behavior. X-ray photoelectron spectroscopy, a powerful photoemission spectroscopy method, generates electron energy spectra by irradiating materials with X-rays, enabling detailed information on elemental composition and chemical bonding states 82,83].

It is essential to emphasize the role of surface characterization in the development of NP-based therapies. Since the surface of NPs is the primary interface with biological systems, its properties directly influence the formation of the protein corona, ligand-receptor binding, circulation time, and even the overall toxicity of the nanocarrier system. Thorough surface characterization, therefore, is not a peripheral task but a central component of NP design and translational potential.

Translational challenges and future directions

Over the last decade, there has been an increased interest towards GBM targeting and considerable advancements have been made in the development of surface-modified NPs, providing them with leadership in this field. Considering the role of BBB in preventing the success of most anti-tumoral therapies, new GBM-targeting studies do not focus only on GBM itself but attempt to improve the rate of BBB crossing as well. However, they still lack specificity within the CNS and can lead to toxic effects to local healthy cells. The only solution would appear to be co-targeting both GBM ligands, to improve selectivity and safety profile, and BBB ligands, to improve the delivery [27, 81, 82]. Dual-targeting daunorubicin liposomes were developed by conjugating with p-aminophenyl-alpha-D-manno-pyranoside and transferrin for transporting drug across the BBB and then targeting glioma cells. This in vitro study showed an increasement up to 24.9% in the transport ratio through the BBB, confirming the interest towards dual-target therapy in GBM treatment [82]. Another more recent in vitro study developed a pH-sensitive dual-targeting drug carrier, G4-DOX-PEG-Tf-TAM, and conjugated it with Transferrin in the exterior and Tamoxifen in the interior of the fourth generation PAMAM dendrimers. Results exhibited a higher BBB transportation ability, with the transporting ratio of 6.06% in 3 h [83]. One more in vivo study tested a novel

UNCORRECTED PROOF

dual-targeting liposomal carrier, incorporated with Tamoxifen and conjugated on their surface with wheat germ agglutinin. Topotecan was then loaded in liposomes. Both in vitro and in vivo results showed a beneficial effect and encouraged further developments in this direction [84]. However, finding two ligands, characterizing them and simultaneously testing their interactions still represents a challenge that will require more research and more studies before becoming a consolidated part of GBM therapy.

All these nanocarrier–drug systems, the so called "Trojan horse complexes," [85, 86] serve as delivery devices that transport specific drugs through the brain endothelium and, subsequently, release it at the appropriate site [65]. This local treatment, compared to systemic ones, would not only improve the efficiency of the anti-tumor drugs, concentrating it right where it is needed, but would also reduce toxicity effects [87]. This does not mean that NPs lack adverse effects. The most common ones are myelotoxicity, vomiting or nausea and Palmoplantar Erythrodysesthesia. Among the rarer, pulmonary embolism, cerebral edema, pneumonia, mucositis and hypophosphatemia represent the most life-threatening ones [88]. It is important to underline that basically no therapeutical gesture in medicine exempt from side effects, including life threatening ones, however, compared to traditional therapies against GBM, NPs seem to have a controlled dose of adverse effects [89]. However, longer studies will be required to exclude long terms complications as well carcinogenicity. The possibility that the use of nanomaterials could lead to genetic alterations and nucleic acids abnormalities was confirmed by Zhang et al. and Singh et al., hence this aspect will need further studies in time [81, 87, 90].

Despite promising results, challenges remain in translating success from in vitro and animal models to human patients. For example, a Phase II study of PEGylated liposomal doxorubicin with temozolomide and radiotherapy for GBM found the treatment to be feasible and safe but did not meaningfully improve patient outcomes [87]. Most experiments rely on in vitro models or animal studies. Recent research tried to develop a human-relevant GBM models that could be used to investigate the efficiency of NPs in an environment as close as possible to the in vivo human one [91–94]. They represent an efficient temporary solution, substituting animal models and avoiding ethical concerns that would arise in in vivo studies. However, as for now, they still present some limitations related to their inability to recreate the temporal and spatial complexity of GBM that make them impossible to be considered a substitute for human testing [95, 96].

Finally, the challenge still represented by surface characterization prevents further advancements, requiring more studies to understand better these new technologies. Researchers, technicians, and industrialists should cooperate to explore options and usefully exploit nanotechnology in field experiments [97]. More studies will be needed to detect a universal biomarker too, to focus all the efforts on it [98]. This raises the demand for developing smart arrays (interdigitated electrode system), multiple detection methods, highly sensitive transducers, and microfluidic systems [99–104].

Alongside systemic administration, other locoregional techniques have emerged to improve nanoparticle delivery in glioblastoma, especially in surmounting the limiting characteristics of the blood-brain barrier. Convection-enhanced delivery (CED) facilitates the direct injection of therapeutic drugs into the brain parenchyma, enabling regulated distribution while circumventing systemic circulation. Preclinical and early clinical investigations indicate that CED markedly enhances drug distribution within the tumor and adjacent infiltrative margin, while reducing systemic toxicity [11]. The intrathecal and intratumoral methods offer potential benefits, especially for confined or recurrent glioblastomas. Although these techniques necessitate neurosurgical intervention and entail procedural risks, they provide potential for attaining therapeutic concentrations that systemic administration frequently does not achieve. Comparative assessments of systemic and intratumoral delivery are crucial for establishing the best therapeutic index and enhancing the clinical translation of nanoparticle-based treatments in GBM patients.

Regulatory and translational hurdles

Despite considerable preclinical advancements in the development of nanoparticle platforms for glioblastoma treatment, their translation into clinical practice is hindered by other substantial non-biological obstacles. Thee regulatory approval necessitates extensive data on biodistribution, clearance, long-term toxicity, and interactions with standard-of-care therapies. The regulatory framework is further complicated by the combined diagnostic and therapeutic (theranostic) functions of certain nanosystems, which may activate combination product paths under FDA or EMA regulations.

Clinical studies are essential in closing this translational gap, offering data on pharmacokinetics, tolerability, and therapeutic efficacy in human participants. Several current trials are assessing nanoparticle-based delivery systems in individuals with recurrent or treatment-resistant glioblastoma. Table 2 summarized selected trials, demonstrating the variety of nanocarrier techniques being explored and their translational significance.

While factors including resabsorption, organ-level accumulation, and nanoparticle clearance are crucial for

UNCORRECTED PROOF

Table 2 Selected ongoing clinical trials of nanoparticle-based strategies in glioblastoma

Clinical Trial Identifier	Intervention Name & Description	Phase	Objective	Translational Relevance
NCT02340156	**Nanoliposomal Irinotecan (Nal-IRI)**: lipid-based carrier for CPT-11 in recurrent GBM	I/II	Assess safety, pharmacokinetics, and dose-limiting toxicity	Demonstrates feasibility of lipid nanocarriers for chemotherapeutic delivery in human GBM
NCT03603379	**BIND-014**: PSMA-targeted polymeric nanoparticle delivering docetaxel	II	Evaluate targeting efficacy and imaging capability	Represents theranostic approach enabling real-time tracking of drug distribution and response

long-term safety, a comprehensive pharmacokinetic analysis exceeds the purview of this review and has been thoroughly explored in pertinent dedicated literature.

Combination strategies

Theranostic nanoparticles signify a notable progression in glioblastoma (GBM) treatment, representing a novel research frontier, that integrates diagnostic and therapeutic capabilities inside a singular nanoplatform.

Theranostic nanoparticles are designed to integrate imaging agents including SPIONs (for MRI), radiolabeled tracers (for PET), and NIR fluorophores (for optical imaging), facilitating real-time, non-invasive observation of biodistribution, tumor targeting, and treatment effectiveness [105, 106]. Enzyme-responsive carriers augment imaging capabilities and facilitate drug release in the GBM microenvironment [105]. Dual-modality technologies integrating MRI and fluorescence facilitate tumor identification during surgical procedures and subsequent monitoring [106]. Theranostic technologies enhance early evaluation of treatment response and increase precision neuro-oncology by the integration of targeted delivery, multimodal imaging, and on-demand release [107].

In addition, the incorporation of nanoparticles (NPs) with traditional treatment methods including radiotherapy, hyperthermia, and phototherapy is a potent translational strategy to improve therapeutic effectiveness in glioblastoma. Gold and hafnium oxide nanoparticles exhibit radiosensitizing properties by enhancing radiation-induced DNA damage and increasing the production of reactive oxygen species, consequently reducing the radiation dose required for tumor management [108, 109]. Likewise, superparamagnetic iron oxide nanoparticles (SPIONs) are under investigation for magnetic hyperthermia, wherein alternating magnetic fields generate localized heating of tumors and impair cellular activity [110]. Photothermal and photodynamic therapies (PTT/PDT) are under investigation, utilizing NIR-absorbing nanoparticles linked with photosensitizers to selectively produce thermal ablation or oxidative stress inside the tumor microenvironment [111]. These combinatorial strategies enhance the synergistic interplay between targeted nanodelivery and conventional treatments to address tumor heterogeneity and resistance mechanisms. Numerous preclinical and early-phase clinical investigations have demonstrated that nanoparticle-mediated radiosensitization or photothermal enhancement may potentially improve survival minimizing collateral tissue damage [112, 113]. The integration of multimodal nanocarriers with tumor-specific ligands or immune modulators may significantly improve tumor specificity and immune activation [114]. As these techniques progress towards clinical application, ongoing investigations persist in refining their safety, biodistribution, and dosimetric profiles in neuro-oncology contexts [114].

Conclusions

Nanocarrier-based drug delivery systems offer promising strategies for overcoming the BBB and achieving targeted treatment of GBM. Surface-modified NPs, particularly those utilizing dual-targeting approaches, enhance drug delivery efficacy while minimizing off-target toxicity. However, clinical translation remains limited due to challenges related to targeting specificity, safety, and complex ligand interactions. Advancing GBM therapy will require deeper insights into NP surface properties and the identification of universal biomarkers. Interdisciplinary collaboration will be essential to bridge the gap between preclinical success and clinical application. AQ4

Abbreviations

Ag-NPs	Silver Nanoparticles
ALMWP	Activatable Low Molecular Weight Protamine
Au-NPs	Gold Nanoparticles
BBB	Blood Brain Barrier
CPP	Cell Penetrating Peptides
CNTs	Carbon Nanotubes
CNS	Central Nervous System
DDS	Drug Delivery System
EGF	Epidermal Growth Factor
EPR	Enhanced Permeation and Retention
FDA	Food and Drug Administration
GBM	Glioblastoma Multiforme

UNCORRECTED PROOF

GSCs	Glioblastoma Stem Cells
HIF	Hypoxia-Inducible-Factor
Lf	Lactoferrin
LDL	Low-Density Lipoprotein
LRP	Lipoprotein-Receptor Related Protein
MMP	Matrix Metalloproteases
NPs	Nanoparticles
PEG-PLA	Poly ethylene glycol-poly lactic acid
PC	Protein Corona
PTX	Paclitaxel
RGD	Arg-Gly-Asp
Se-NPs	Selenium Nanoparticles
TAMs	Tumor Associated Macrophages
TANs	Tumor-Associated Nerves
TME	Tumor Microenvironment
Tf	Transferrin
VEGF	Vascular Endothelial Growth Factor
WHO	World Health Organization

Acknowledgements Not applicable.

Author contributions Conceptualization, G.D.R., E.A., M.Z., M.M.F., and P.P.P.; methodology, G.D.R., M.Z., A.T., P.P.P., M.M.F., T.I., and E.A. validation, G.D.R., M.Z., A.T., C.G., D.T., P.P.P., M.M.F., T.I., and E.A. formal analysis, G.D.R., P.P.P., M.M.F., T.I., and E.A.; investigation, G.D.R., M.Z., A.T., C.G., D.T., P.P.P., M.M.F., T.I., and E.A.; resources, E.A., and M.Z.; data curation, G.D.R., M.Z., A.T., C.G., D.T., P.P.P., M.M.F., T.I., and E.A.; writing-original draft preparation, E.A.; writing-review and editing, G.D.R., M.Z., A.T., C.G., D.T., P.P.P., M.M.F., T.I., and E.A.; visualization, G.D.R., M.Z., A.T., C.G., D.T., P.P.P., M.M.F., T.I., and E.A..; supervision, E.A., M.M.F., and P.P.P.; project administration, E.A., M.Z., M.M.F., and P.P.P.;. All a[illegible]thors have read and agreed to the published version of the manus[illegible]pt.

Funding This research received no external funding.

Declarations

Institutional Review Board Statement Not a[illegible]icable.

Informed Consent Statement Not app[illegible]able.

Conflict of interest Nothing to d[illegible]lare.

References

1. Wirschin[illegible]G, Galanis E, Weller M (2016) Glioblastoma. Handb Clin N[illegible]rol 134:381–397. https://doi.org/10.1016/B978-0-12-80299[illegible]8.00023-2
2. [illegible]mple RC, Bhargava S, Dixit D, Rich JN (2019) Glioblastoma stem cells: lessons from the tumor hierarchy in a lethal cancer. Genes Dev 33(11–12):591–609. https://doi.org/10.1101/gad.324301.119
3. Tan AC, Ashley DM, López GY, Malinzak M, Friedman HS, Khasraw M (2020) Management of glioblastoma: state of the art and future directions. Cancer J Clin 70(4):299–312. https://doi.org/10.3322/caac.21613
4. Delgado-López PD, Corrales-García EM (2016) Survival in glioblastoma: a review on the impact of treatment modalities. Clin Translational Oncology: Official Publication Federation Span Oncol Soc Natl Cancer Inst Mexico 18(11):1062–1071. https://doi.org/10.1007/s12094-016-1497-x
5. Louis DN, Perry A, Wesseling P, Brat DJ, Cree IA, Figarella-Branger D, Hawkins C, Ng HK, Pfister SM, Reifenberger G, Soffietti R, von Deimling A, Ellison DW (2021) The 2021 WHO classification of tumors of the central nervous system: a summary. Neuro-Oncol 23(8):1231–1251. https://doi.org/10.1093/neuonc/noab106
6. Smith HL, Wadhwani N, Horbinski C (2022) Major features of the 2021 WHO classification of CNS tumors. Neurotherap[illegible]ics 19(6):1691–1704. https://doi.org/10.1007/s13311-022-01[illegible]9-0
7. Stupp R, Mason WP, van den Bent MJ, Weller M, [illegible]isher B, Taphoorn MJ, Belanger K, Brandes AA, Marosi[illegible]C, Bogdahn U, Curschmann J, Janzer RC, Ludwin SK, Gor[illegible]T, Allgeier A, Lacombe D, Cairncross JG, Eisenhauer E, Mi[illegible]manoff RO, European Organisation for Research and Trea[illegible]ent of Cancer Brain Tumor and Radiotherapy Groups, Na[illegible]nal Cancer Institute of Canada Clinical Trials Group (2005) [illegible]adiotherapy plus concomitant and adjuvant temozolomide [illegible] glioblastoma. N Engl J Med 352(10):987–996. https://doi.[illegible]/10.1056/NEJMoa043330
8. Lakomy R, Kazda T, Selin[illegible]ova I, Poprach A, Pospisil P, Belanova R, Fadrus P, Vybi[illegible]V, Smrcka M, Jancalek R, Hynkova L, Muckova K, Hend[illegible]ch M, Sana J, Slaby O, Slampa P (2020) Real-World evide[illegible]e in glioblastoma: stupp's regimen after a decade. Front O[illegible]col 10:840. https://doi.org/10.3389/fonc.2020.00840
9. Jain KK ([illegible]18) A critical overview of targeted therapies for glioblasto[illegible]. Front Oncol 8:419. https://doi.org/10.3389/fonc.2018.004[illegible]
10. M[illegible]nini I, Caponnetto F, Dalla E, Ius T, Della Pepa GM, Pegolo E, Bartolini A, La Rocca G, Menna G, Di Loreto C, Olivi A, Skrap M, Sabatino G, Cesselli D (2020) Heterogeneity matters: different regions of glioblastoma are characterized by distinctive Tumor-Supporting pathways. Cancers 12(10):2960. https://doi.org/10.3390/cancers12102960
11. Ius T, Somma T, Pasqualetti F, Berardinelli J, Vitulli F, Caccese M, Cella E, Cenciarelli C, Pozzoli G, Sconocchia G, Zeppieri M, Gerardo C, Caffo M, Lombardi G (2024) Local therapy in glioma: an evolving paradigm from history to horizons (Review). Oncol Lett 28(3):440. https://doi.org/10.3892/ol.2024.14573
12. Ius T, Sabatino G, Panciani PP, Fontanella MM, Rudà R, Castellano A, Barbagallo GMV, Belotti F, Boccaletti R, Catapano G, Costantino G, Della Puppa A, Di Meco F, Gagliardi F, Garbossa D, Germanò AF, Iacoangeli M, Mortini P, Olivi A, Pessina F, Esposito V (2023) Surgical management of Glioma Grade 4: technical update from the neuro-oncology section of the Italian Society of Neurosurgery (SINch®): a systematic review. J of neuro-oncol 162(2):267–293. https://doi.org/10.1007/s11060-023-04274-x
13. Ballabh P, Braun A, Nedergaard M (2004) The blood-brain barrier: an overview: structure, regulation, and clinical implications. Neurobiol Dis 16(1):1–13. https://doi.org/10.1016/j.nbd.2003.12.016
14. Daneman R, Prat A (2015) The blood-brain barrier. Cold Spring Harb Perspect Biol 7(1):a020412. https://doi.org/10.1101/cshperspect.a020412
15. Dixit S, Novak T, Miller K, Zhu Y, Kenney ME, Broome AM (2015) Transferrin receptor-targeted theranostic gold nanoparticles for photosensitizer delivery in brain tumors. Nanoscale 7(5):1782–1790. https://doi.org/10.1039/c4nr04853a
16. Ulbrich K, Knobloch T, Kreuter J (2011) Targeting the insulin receptor: nanoparticles for drug delivery across the blood-brain

UNCORRECTED PROOF

barrier (BBB). J Drug Target 19(2):125–132. https://doi.org/10.3109/10611861003734001
17. He C, Li J, Cai P, Ahmed T, Henderson J, Foltz W, Bendayan Reina, Rauth Andrew, Wu XY (2018) Two-step targeted hybrid nanoconstructs increase brain penetration and efficacy of the therapeutic antibody trastuzumab against brain metastasis of HER2-positive breast cancer. Adv Funct Mater 28:1705668. https://doi.org/10.1002/adfm.201705668
18. Qiao R, Jia Q, Hüwel S, Xia R, Liu T, Gao F, Galla HJ, Gao M (2012) Receptor-mediated delivery of magnetic nanoparticles across the blood-brain barrier. ACS Nano 6(4):3304–3310. https://doi.org/10.1021/nn300240p
19. Wei X, Zhan C, Shen Q, Fu W, Xie C, Gao J, Peng C, Zheng P, Lu W (2015) A d-peptide ligand of nicotine acetylcholine receptors for brain-targeted drug delivery. Angew Chem Int Ed Engl 54(10):3023–3027. https://doi.org/10.1002/anie.201411226
20. Jiang X, Xin H, Ren Q, Gu J, Zhu L, Du F, Feng C, Xie Y, Sha X, Fang X (2014) Nanoparticles of 2-deoxy-D-glucose functionalized poly(ethylene glycol)-co-poly(trimethylene carbonate) for dual-targeted drug delivery in glioma treatment. Biomaterials 35(1):518–529. https://doi.org/10.1016/j.biomaterials.2013.09.094
21. Martins C, Sarmento B (2023) Multi-ligand functionalized blood-to-tumor sequential targeting strategies in the field of glioblastoma nanomedicine. WIREs Nanomed Nanobiotechnol 15(5):e1893. https://doi.org/10.1002/wnan.1893
22. Gao JQ, Lv Q, Li LM, Tang XJ, Li FZ, Hu YL, Han M (2013) Glioma targeting and blood-brain barrier penetration by dual-targeting doxorubincin liposomes. Biomaterials 34(22):5628–5639. https://doi.org/10.1016/j.biomaterials.2013.03.097
23. Dotiwala AK, McCausland C, Samra NS (2023) Anatomy, head and neck: blood brain barrier. StatPearls. StatPearls Publishing
24. Weathers SP, de Groot J (2015) VEGF manipulation in glioblastoma. Oncol (Williston Park N Y) 29(10):720–727
25. Yi Y, Hsieh IY, Huang X, Li J, Zhao W (2016) Glioblastoma stem-like cells: characteristics, microenvironment, and therapy. Front Pharmacol 7:477. https://doi.org/10.3389/fphar.2016.00477
26. Pavon LF, Marti LC, Sibov TT, Malheiros SM, Brandt RA, Cavalheiro S, Gamarra LF (2014) In vitro analysis of neurospheres derived from glioblastoma primary culture: a novel methodology paradigm. Front Neurol 4:214. https://doi.org/10.3389/fneur.2013.00214
27. Liebelt BD, Shingu T, Zhou X, Ren J, Shin SA, Hu J (2016) Glioma Stem Cells: Signaling, Microenvironment, and Therapy. *Stem cells international*, *2016*, 7849890. https://doi.org/10.1155/2016/7849890
28. Brescia P, Ortensi B, Fornasari L, Levi D, Broggi G, Pelicci G (2013) CD133 is essential for glioblastoma stem cell maintenance. Stem Cells 31(5):857–869. https://doi.org/10.1002/stem.1317
29. Shibahara I, Sonoda Y, Saito R, Kanamori M, Yamashita Y, Kumabe T, Watanabe M, Suzuki H, Watanabe T, Ishioka C, Tominaga T (2013) The expression status of CD133 is associated with the pattern and timing of primary glioblastoma recurrence. Neuro Oncol 15(9):1151–1159. https://doi.org/10.1093/neuonc/not066
30. Han M, Guo L, Zhang Y, Huang B, Chen A, Chen W, Liu X, Sun S, Wang K, Liu A, Li X (2016) Clinicopathological and prognostic significance of CD133 in glioma patients: a meta-analysis. Mol Neurobiol 53(1):720–727. https://doi.org/10.1007/s12035-014-9018-9
31. Yan X, Ma L, Yi D, Yoon JG, Diercks A, Foltz G, Price ND, Hood LE, Tian Q (2011) A CD133-related gene expression signature identifies an aggressive glioblastoma subtype with excessive mutations. Proc Natl Acad Sci U S A 108(4):1591–1596. https://doi.org/10.1073/pnas.1018696108
32. Nayak A, Warrier N, Raman R, Prabhu V, Kumar P (2024) Targeted delivery of nanomedicines to glioblastoma: overcoming the clinical barrier. J Drug Deliv Sci Technol 105980. 99https://doi.org/10.1016/j.jddst.2024.105980
33. Mao JM, Liu J, Guo G, Mao XG, Li CX (2015) Glioblastoma vasculogenic mimicry: signaling pathways progression and potential anti-angiogenesis targets. Biomark Res 3:8. https://doi.org/10.1186/s40364-015-0034-3
34. Shi Y, van der Meel R, Chen X, Lammers T (2020) The EPR effect and beyond: strategies to improve tumor targeting and cancer nanomedicine treatment efficacy. Theranostics 10(17):7921–7924. https://doi.org/10.7150/thno.49577
35. Danhier F (2016) To exploit the tumor microenvironment: since the EPR effect fails in the clinic, what is the future of nanomedicine? J Controlled Release: Official J Controlled Release Soc 244(Pt A):108–121. https://doi.org/10.1016/j.jconrel.2016.11.015
36. Nakamura Y, Mochida A, Choyke PL, Kobayashi H (2016) Nanodrug delivery: is the enhanced permeability and retention effect sufficient for curing cancer?? Bioconjug Chem 27(10):2225–2238. https://doi.org/10.1021/acs.bioconjchem.6b00437
37. de Lázaro I, Mooney DJ (2020) A nanoparticle's pathway into tumours. Nat Mater 19(5):486–487. https://doi.org/10.1038/s41563-020-0669-9
38. Golombek SK, May JN, Theek B, Appold L, Drude N, Kiessling F, Lammers T (2018) Tumor targeting via EPR: strategies to enhance patient responses. Adv Drug Deliv Rev 130:17–38. https://doi.org/10.1016/j.addr.2018.07.007
39. Lv Y, Xu C, Zhao X, Lin C, Yang X, Xin X, Zhang L, Qin C, Han X, Yang L, He W, Yin L (2018) Nanoplatform assembled from a CD44-targeted prodrug and smart liposomes for dual targeting of tumor microenvironment and cancer cells. ACS Nano 12(2):1519–1536. https://doi.org/10.1021/acsnano.7b08051
40. Ambasta RK, Sharma A, Kumar P (2011) Nanoparticle mediated targeting of VEGFR and cancer stem cells for cancer therapy. Vasc Cell 3:26. https://doi.org/10.1186/2045-824X-3-26
41. Gysler SM, Drapkin R (2021) Tumor innervation: peripheral nerves take control of the tumor microenvironment. J Clin Invest 131(11):e147276. https://doi.org/10.1172/JCI147276
42. Zahalka AH, Arnal-Estapé A, Maryanovich M, Nakahara F, Cruz CD, Finley LWS, Frenette PS (2017) Adrenergic nerves activate an angio-metabolic switch in prostate cancer. Science 358(6361):321–326. https://doi.org/10.1126/science.aah5072
43. Ahlawat J, Guillama Barroso G, Masoudi Asil S, Alvarado M, Armendariz I, Bernal J, Carabaza X, Chavez S, Cruz P, Escalante V, Estorga S, Fernandez D, Lozano C, Marrufo M, Ahmad N, Negrete S, Olvera K, Parada X, Portillo B, Ramirez A, Ramos Raul, Rodriguez Veronica, Rojas Paola, Romero Jessica, Suarez David, Urueta Graciela, Viel Stephanie, Narayan M (2020) Nanocarriers as potential drug delivery candidates for overcoming the blood-brain barrier: challenges and possibilities. ACS omega 5(22):12583–12595. https://doi.org/10.1021/acsomega.0c01592
44. Farooq M, Scalia G, Umana GE, Parekh UA, Naeem F, Abid SF, Khan MH, Zahra SG, Sarkar HP, Chaurasia B (2023) A systematic review of nanomedicine in glioblastoma treatment: clinical efficacy, safety, and future directions. Brain Sci 13(12):1727. https://doi.org/10.3390/brainsci13121727
45. Khan I, Baig MH, Mahfooz S, Imran MA, Khan MI, Dong JJ, Cho JY, Hatiboglu MA (2022) Nanomedicine for glioblastoma: progress and future prospects. Sem Cancer Biol 86(Pt 2):172–186. https://doi.org/10.1016/j.semcancer.2022.06.007
46. Dang Y, Guan J (2020) Nanoparticle-based drug delivery systems for cancer therapy. Smart Mater Med 1:10–19. https://doi.org/10.1016/j.smaim.2020.04.001

UNCORRECTED PROOF

47. Anselmo AC, Mitragotri S (2016) Nanoparticles in the clinic. Bioeng Transl Med 1(1):10–29. https://doi.org/10.1002/btm2.10003
48. Amaral M, Cruz N, Rosa A, Nogueira B, Costa D, Santos F, Brazão M, Policarpo P, Mateus R, Kobozev Y, Reis CP (2021) An update of advanced nanoplatforms for glioblastoma multiforme management. EXCLI J 20:1544–1570. https://doi.org/10.17179/excli2021-4393
49. Di Filippo LD, Duarte JL, Luiz MT, de Araújo JTC, Chorilli M (2021) Drug delivery nanosystems in glioblastoma multiforme treatment: current state of the art. Curr Neuropharmacol 19(6):787–812. https://doi.org/10.2174/1570159X18666200831160627
50. Liu D, Dai X, Ye L, Wang H, Qian H, Cheng H, Wang X (2023) Nanotechnology meets glioblastoma multiforme: emerging therapeutic strategies. WIREs Nanomed Nanobiotechnol 15(1):e1838. https://doi.org/10.1002/wnan.1838
51. Zhang N, Wei L, Ye M, Kang C, You H (2020) Treatment progress of immune checkpoint blockade therapy for glioblastoma. Front Immunol 11:592612. https://doi.org/10.3389/fimmu.2020.592612
52. Wang S, Yang L, He W, Zheng M, Zou Y (2025) Cell membrane camouflaged biomimetic nanoparticles as a versatile platform for brain diseases treatment. Small Methods 9(1):e2400096. https://doi.org/10.1002/smtd.202400096
53. Jena L, McErlean E, McCarthy H (2020) Delivery across the blood-brain barrier: nanomedicine for glioblastoma multiforme. Drug Deliv Transl Res 10(2):304–318. https://doi.org/10.1007/s13346-019-00679-2
54. Li J, Zhao J, Tan T, Liu M, Zeng Z, Zeng Y, Zhang L, Fu C, Chen D, Xie T (2020) Nanoparticle drug delivery system for glioma and its efficacy improvement strategies: a comprehensive review. Int J Nanomed 15:2563–2582. https://doi.org/10.2147/IJN.S243223
55. Liu Z, Ji X, He D, Zhang R, Liu Q, Xin T (2022) Nanoscale drug delivery systems in glioblastoma. Nanoscale Res Lett 17(1):27. https://doi.org/10.1186/s11671-022-03668-6
56. Hersh AM, Alomari S, Tyler BM (2022) Crossing the blood-brain barrier: advances in nanoparticle technology for drug delivery in neuro-oncology. Int J Mol Sci 23(8):4153. https://doi.org/10.3390/ijms23084153
57. Treuel L, Nienhaus GU (2012) Toward a molecular understanding of nanoparticle-protein interactions. Biophys Rev 4(2):137–147. https://doi.org/10.1007/s12551-012-0072-0
58. Huang W, Xiao G, Zhang Y, Min W (2021) Research progress and application opportunities of nanoparticle-protein corona complexes. Biomed Pharmacother 139:111541. https://doi.org/10.1016/j.biopha.2021.111541
59. Gao X, Tao W, Lu W, Zhang Q, Zhang Y, Jiang X, Fu S (2006) Lectin-conjugated PEG-PLA nanoparticles: preparation and brain delivery after intranasal administration. Biomaterials 27(18):3482–3490. https://doi.org/10.1016/j.biomaterials.2006.01.038
60. Gu G, Xia H, Hu Q, Liu Z, Jiang M, Kang T, Miao D, Tu Y, Pang Z, Song Q, Yao L, Chen H, Gao X, Chen J (2013) PEG-co-PCL nanoparticles modified with MMP-2/9 activatable low molecular weight protamine for enhanced targeted glioblastoma therapy. Biomaterials 34(1):196–208. https://doi.org/10.1016/j.biomaterials.2012.09.044
61. AbdEl-Haq M, Kumar A, Ait Mohand FE, Kravchenko-Balasha N, Rottenberg Y, Domb AJ (2023) Paclitaxel delivery to the brain for glioblastoma treatment. Int J Mol Sci 24(14):11722. https://doi.org/10.3390/ijms241411722
62. Visser CC, Stevanović S, Heleen Voorwinden L, Gaillard PJ, Crommelin DJ, Danhof M, De Boer AG (2004) Validation of the transferrin receptor for drug targeting to brain capillary endothelial cells in vitro. J Drug Target 12(3):145–150. https://doi.org/10.1080/10611860410001701706
63. Ulbrich K, Hekmatara T, Herbert E, Kreuter J (2009) Transferrin- and transferrin-receptor-antibody-modified nanoparticles enable drug delivery across the blood-brain barrier (BBB). Eur J Pharm Biopharmaceutics: Official J Arbeitsgemeinschaft Fur Pharmazeutische Verfahrenstechnik E V 71(2):251–256. https://doi.org/10.1016/j.ejpb.2008.08.021
64. Paris-Robidas S, Emond V, Tremblay C, Soulet D, Calon F (2011) In vivo labeling of brain capillary endothelial cells after intravenous injection of monoclonal antibodies targeting the transferrin receptor. Mol Pharmacol 80(1):32–39. https://doi.org/10.1124/mol.111.071027
65. Moos T, Morgan EH (2001) Restricted transport of anti-transferrin receptor antibody (OX26) through the blood-brain barrier in the rat. J Neurochem 79(1):119–129. https://doi.org/10.1046/j.1471-4159.2001.00541.x
66. Gosk S, Vermehren C, Storm G, Moos T (2004) Targeting anti-transferrin receptor antibody (OX26) and OX26-conjugated liposomes to brain capillary endothelial cells using in situ perfusion. J Cereb Blood Flow Metabolism: Official J Int Soc Cereb Blood Flow Metabolism 24(11):1193–1204. https://doi.org/10.1097/01.WCB.0000135592.28823.47
67. Georgieva JV, Hoekstra D, Zuhorn IS (2014) Smuggling drugs into the brain: an overview of ligands targeting transcytosis for drug delivery across the blood-brain barrier. Pharmaceutics 6(4):557–583. https://doi.org/10.3390/pharmaceutics6040557
68. Demeule M, Poirier J, Jodoin J, Bertrand Y, Desrosiers RR, Dagenais C, Nguyen T, Lanthier J, Gabathuler R, Kennard M, Jefferies WA, Karkan D, Tsai S, Fenart L, Cecchelli R, Béliveau R (2002) High transcytosis of melanotransferrin (P97) across the blood-brain barrier. J Neurochem 83(4):924–933. https://doi.org/10.1046/j.1471-4159.2002.01201.x
69. Boison D (2008) Adenosine as a neuromodulator in neurological diseases. Curr Opin Pharmacol 8(1):2–7. https://doi.org/10.1016/j.coph.2007.09.002
70. Stone TW, Ceruti S, Abbracchio MP (2009) Adenosine receptors and neurological disease: neuroprotection and neurodegeneration. Handb Exp Pharmacol 193:535–587. https://doi.org/10.1007/978-3-540-89615-9_17
71. Möser GH, Schrader J, Deussen A (1989) Turnover of adenosine in plasma of human and dog blood. Am J Physiol 256(4):C799–C806. https://doi.org/10.1152/ajpcell.1989.256.4.C799
72. Gao X, Qian J, Zheng S, Changyi Y, Zhang J, Ju S, Zhu J, Li C (2014) Overcoming the blood-brain barrier for delivering drugs into the brain by using adenosine receptor nanoagonist. ACS Nano 8(4):3678–3689. https://doi.org/10.1021/nn5003375
73. Gaudin A, Yemisci M, Eroglu H, Lepetre-Mouelhi S, Turkoglu OF, Dönmez-Demir B, Caban S, Sargon MF, Garcia-Argote S, Pieters G, Loreau O, Rousseau B, Tagit O, Hildebrandt N, Le Dantec Y, Mougin J, Valetti S, Chacun H, Nicolas V, Desmaële D, Couvreur P (2014) Squalenoyl adenosine nanoparticles provide neuroprotection after stroke and spinal cord injury. Nat nanotechnol 9(12):1054–1062. https://doi.org/10.1038/nnano.2014.274
74. Liu W, Su J, Shi Q, Wang J, Chen X, Zhang S, Li M, Cui J, Fan C, Sun B, Wang G (2021) RGD peptide-conjugated selenium nanocomposite inhibits human glioma growth by triggering mitochondrial dysfunction and ROS-dependent MAPKs activation. Front Bioeng Biotechnol 9:781608. https://doi.org/10.3389/fbioe.2021.781608
75. Hosseinikhah SMVahdat lasemi, FatemehFarhoudi, LeilaGupta, GarimaKesharwani, Prashant, Sahebkar A (2024) RGD-decorated nanoparticles: Therapeutic potential beyond cancer. Journal of Drug Delivery Science and Technology. 98. 105924. https://doi.org/10.1016/j.jddst.2024.105924

UNCORRECTED PROOF

76. Montazersaheb S, Eftekhari A, Shafaroodi A, Tavakoli S, Jafari S, Baran Ayşe, Baran Mehmet, Jafari Sevda, Ahmadian Elham (2024) Green-synthesized silver nanoparticles from peel extract of pumpkin as a potent radiosensitizer against triple-negative breast cancer (TNBC). Cancer Nanotechnol. https://doi.org/10.1186/s12645-024-00285-z
77. Rosic G (2024) Cancer signaling, cell/gene therapy, diagnosis and role of nanobiomaterials. Advances in Biology & Earth Sciences 9:11–34. https://doi.org/10.62476/abes9s11
78. Gunes, Muslum & Ertaş, Erdal & Seyhmus, Tumur & Zulfugarova, Parvin & Nuriyeva, Fidan& Kavetskyy, Taras & Kukhazh, Yuliia & Grozdov, Pavlo & Šauša, O. & Smutok, Oleh &Ganbarov, Dashgin & Kiv, Arnold. (2025). Synthesis and Antibacterial Evaluation of Silver-Coated Magnetic Iron Oxide/Activated Carbon Nanoparticles Derived from Hibiscus esculentus. DOI:https://doi.org/10.20944/preprints202505.1245.v1
79. Seçkin Hamdullah, Meydan İsmet (2021) Synthesis and characterization of *Veronica beccabunga* green synthesized silver nanoparticles for the antioxidant and antimicrobial activity. Türkiye Tarımsal Araştırmalar Dergisi. https://doi.org/10.19159/tutad.805463
80. Baer DR, Engelhard MH, Johnson GE, Laskin J, Lai J, Mueller K, Munusamy P, Thevuthasan S, Wang H, Washton N, Elder A, Baisch BL, Karakoti A, Kuchibhatla SV, Moon D (2013) Surface characterization of nanomaterials and nanoparticles: Important needs and challenging opportunities. *Journal of vacuum science & technology. A*, Vacuum, *surfaces, and films: an official journal of the American Vacuum Society*, *31*(5), 50820. https://doi.org/10.1116/1.4818423
81. Rodà F, Caraffi R, Picciolini S, Tosi G, Vandelli MA, Ruozi B, Bedoni M, Ottonelli I, Duskey JT (2023) Recent advances on surface-modified GBM targeted nanoparticles: targeting strategies and surface characterization. Int J Mol Sci 24(3):2496. https://doi.org/10.3390/ijms24032496
82. Ying X, Wen H, Lu WL, Du J, Guo J, Tian W, Men Y, Zhang Y, Li RJ, Yang TY, Shang DW, Lou JN, Zhang LR, Zhang Q (2010) Dual-targeting daunorubicin liposomes improve the therapeutic efficacy of brain glioma in animals. J Controlled Release: Official J Controlled Release Soc 141(2):183–192. https://doi.org/10.1016/j.jconrel.2009.09.020
83. Li Y, He H, Jia X, Lu WL, Lou J, Wei Y (2012) A dual-targeting nanocarrier based on poly(amidoamine) dendrimers conjugated with transferrin and tamoxifen for treating brain gliomas. Biomaterials 33(15):3899–3908. https://doi.org/10.1016/j.biomaterials.2012.02.004
84. Du J, Lu WL, Ying X, Liu Y, Du P, Tian W, Men Y, Guo J, Zhang Y, Li RJ, Zhou J, Lou JN, Wang JC, Zhang X, Zhang Q (2009) Dual-targeting Topotecan liposomes modified with Tamoxifen and wheat germ agglutinin significantly improve drug transport across the blood-brain barrier and survival of brain tumor-bearing animals. Mol Pharm 6(3):905–917. https://doi.org/10.1021/mp800218q
85. Zhang L, Zhang Y, Wang X, Zhou Y, Qi J, Gu L, Zhao Q, Yu R, Zhou X (2023) A Trojan-Horse-Like biomimetic Nano-NK to elicit an immunostimulatory tumor microenvironment for enhanced GBM Chemo-Immunotherapy. Small 19(44):e2301439. https://doi.org/10.1002/smll.202301439
86. Nelson D, Fisher S, Robinson B (2014) The trojan horse approach to tumor immunotherapy: targeting the tumor microenvironment. J Immunol Res 2014:789069. https://doi.org/10.1155/2014/789069
87. Qian L, Zheng J, Wang K, Tang Y, Zhang X, Zhang H, Huang F, Pei Y, Jiang Y (2013) Cationic core-shell nanoparticles with carmustine contained within O^6-benzylguanine shell for glioma therapy. Biomaterials 34(35):8968–8978. https://doi.org/10.1016/j.biomaterials.2013.07.097
88. Duan M, Cao R, Yang Y, Chen X, Liu L, Ren B, Wang L, Goh BC (2024) Blood-brain barrier conquest in glioblastoma nanomedicine: strategies, clinical advances, and emerging challenges. Cancers 16(19):3300. https://doi.org/10.3390/cancers16193300
89. Krishna Y, Fauzan, Ir Ts Dr Mohd Faizal &, Saidur R, Ng KC, Aslfattahi N (2020) State-of-the-art heat transfer fluids for parabolic trough collector. Int J Heat Mass Transf 152:119541. https://doi.org/10.1016/j.ijheatmasstransfer.2020.119541
90. Beier CP, Schmid C, Gorlia T, Kleinletzenberger C, Beier D, Grauer O, Steinbrecher A, Hirschmann B, Brawanski A, Dietmaier C, Jauch-Worley T, Kölbl O, Pietsch T, Proescholdt M, Rümmele P, Muigg A, Stockhammer G, Hegi M, Bogdahn U, Hau P (2009) RNOP-09: pegylated liposomal doxorubicine and prolonged temozolomide in addition to radiotherapy in newly diagnosed glioblastoma–a phase II study. BMC Cancer 9:308. https://doi.org/10.1186/1471-2407-9-308
91. Bezze A, Ciardelli G, Mattu C (2023) In vitro human-relevant glioblastoma models as the novel frontier of nanomedicine screening. Biomedical Science and Engineering 4 https://doi.org/10.4081/bse.222
92. Schulz JA, Rodgers LT, Kryscio RJ, Hartz AMS, Bauer B (2022) Characterization and comparison of human glioblastoma models. BMC Cancer 22(1):844. https://doi.org/10.1186/s12885-022-09910-9
93. Tatla AS, Justin AW, Watts C, Markaki AE (2021) A vascularized tumoroid model for human glioblastoma angiogenesis. Sci Rep 11(1):19550. https://doi.org/10.1038/s41598-021-98911-y
94. Gómez-Oliva R, Domínguez-García S, Carrascal L, Abalos-Martínez J, Pardillo-Díaz R, Verástegui C, Castro C, Nunez-Abades P, Geribaldi-Doldán N (2021) Evolution of experimental models in the study of glioblastoma: toward finding efficient treatments. Front Oncol 10:614295. https://doi.org/10.3389/fonc.2020.614295
95. Zhao C, Zhu X, Tan J, Mei C, Cai X, Kong F (2024) Lipid-based nanoparticles to address the limitations of GBM therapy by overcoming the blood-brain barrier, targeting glioblastoma stem cells, and counteracting the immunosuppressive tumor microenvironment. Biomed Pharmacother 171:116113. https://doi.org/10.1016/j.biopha.2023.116113
96. Stanković T, Ranđelović T, Dragoj M, Stojković Burić S, Fernández L, Ochoa I, Pérez-García VM, Pešić M (2021) In vitro biomimetic models for glioblastoma-a promising tool for drug response studies. Drug Resist Updat 55:100753. https://doi.org/10.1016/j.drup.2021.100753
97. Malik S, Muhammad K, Waheed Y (2023) Nanotechnology: a revolution in modern industry. Molecules 28(2):661. https://doi.org/10.3390/molecules28020661
98. Ding W, Zhou X, Jiang G, Xu W, Long S, Xiao F, Liao Y, Liu J (2022) Identification of prognostic biomarkers of glioblastoma based on multidatabase integration and its correlation with immune-infiltration cells. J Oncol 2022(3909030). https://doi.org/10.1155/2022/3909030
99. Kaushik Ajeet (2019) Biomedical nanotechnology related grand challenges and perspectives. Frontiers in Nanotechnology. https://doi.org/10.3389/fnano.2019.00001
100. Hu D, Xia M, Wu L, Liu H, Chen Z, Xu H, He C, Wen J, Xu X (2023) Challenges and advances for glioma therapy based on inorganic nanoparticles. Mater Today Bio 20:100673. https://doi.org/10.1016/j.mtbio.2023.100673
101. Sharma K (2015) Book - Nanobiotechnology for sensing applications: From lab to field
102. Gomes M, Ramalho MJ, Loureiro JA, Pereira MC (2025) Advancing brain targeting: cost-effective surface-modified nanoparticles for faster market entry. Pharmaceutics 17(5):661. https://doi.org/10.3390/pharmaceutics17050661

UNCORRECTED PROOF

103. Madani F, Morovvati H, Webster TJ, Najaf Asaadi S, Rezayat SM, Hadjighassem M, Khosravani M, Adabi M (2024) Combination chemotherapy via poloxamer 188 surface-modified PLGA nanoparticles that traverse the blood-brain-barrier in a glioblastoma model. Sci Rep 14(1):19516. https://doi.org/10.1038/s41598-024-69888-1
104. Noorani I, de la Rosa J (2023) Breaking barriers for glioblastoma with a path to enhanced drug delivery. Nat Commun 14:5909. https://doi.org/10.1038/s41467-023-41694-9
105. Chan MH, Chen W, Li CH, Fang CY, Chang YC, Wei DH, Liu RS, Hsiao M (2021) An advanced in situ magnetic resonance imaging and ultrasonic theranostics nanocomposite platform: crossing the Blood-Brain barrier and improving the suppression of glioblastoma using Iron-Platinum nanoparticles in nanobubbles. ACS Appl Mater Interfaces 13(23):26759–26769. https://doi.org/10.1021/acsami.1c04990
106. Zhao X, Zhao H, Chen Z, Lan M (2014) Ultrasmall superparamagnetic iron oxide nanoparticles for magnetic resonance imaging contrast agent. J Nanosci Nanotechnol 14(1):210–220. https://doi.org/10.1166/jnn.2014.9192
107. Zhong X, Wei X, Xu Y, Zhu X, Huo B, Guo X, Feng G, Zhang Z, Feng X, Fang Z, Luo Y, Yi X, Jiang Ding-Sheng (2024) The lysine methyltransferase SMYD2 facilitates neointimal hyperplasia by regulating the HDAC3–SRF axis. Acta Pharm Sin B. https://doi.org/10.1016/j.apsb.2023.11.012
108. Kuo YC, Lin PI, Wang CC (2011) Targeting nevirapine delivery across human brain microvascular endothelial cells using transferrin-grafted poly(lactide-co-glycolide) nanoparticles. Nanomed (London England) 6(6):1011–1026. https://doi.org/10.2217/nnm.11.25
109. Maggiorella L, Barouch G, Devaux C, Pottier A, Deutsch E, Bourhis J, Borghi E, Levy L (2012) Nanoscale radiotherapy with hafnium oxide nanoparticles. Future Oncol (London England) 8(9):1167–1181. https://doi.org/10.2217/fon.12.96
110. Johannsen M, Thiesen B, Jordan A, Taymoorian K, Gneveckow U, Waldöfner N, Scholz R, Koch M, Lein M, Jung K, Loening SA (2005) Magnetic fluid hyperthermia (MFH) reduces prostate cancer growth in the orthotopic dunning R3327 rat model. Prostate 64(3):283–292. https://doi.org/10.1002/pros.20213
111. Lucky SS, Soo KC, Zhang Y (2015) Nanoparticles in photodynamic therapy. Chem Rev 115(4):1990–2042. https://doi.org/10.1021/cr5004198
112. Riley RS, Day ES (2017) Gold nanoparticle-mediated photothermal therapy: applications and opportunities for multimodal cancer treatment. WIREs Nanomed Nanobiotechnol. https://doi.org/10.1002/wnan.1449
113. Camille Verry S, Dufort J, Villa M, Gavard C, Iriart S, Grand J, Charles B, Chovelon J-L, Cracowski J-L, Quesada C, Mendoza L, Sancey A, Lehmann F, Jover J-Y, Giraud PJ, Pauwels Daniel Cagney, Ross Berbeco, Ayal Aizer, Eric Deutsch, Markus Loeffler, Géraldine Le Duc, Olivier Tillement, Jacques Balosso. (2021). Theranostic AGuIX nanoparticles as radiosensitizer: A phase I, dose-escalation study in patients with multiple brain metastases (NANO-RAD trial). *Radiotherapy and Oncology, Volume 160*. https://doi.org/10.1016/j.radonc.2021.04.021
114. Kunjachan S, Ehling J, Storm G, Kiessling F, Lammers T (2015) Noninvasive imaging of nanomedicines and nanotheranostics: principles, progress, and prospects. Chem Rev 115(19):10907–10937. https://doi.org/10.1021/cr500314d
115. Li J, Guo Y, Kuang Y, An S, Ma H, Jiang C (2013) Choline transporter-targeting and co-delivery system for glioma therapy. Biomaterials 34(36):9142–9148. https://doi.org/10.1016/j.biomaterials.2013.08.030
116. Bekas D, Tsirka K, Baltzis D, Paipetis A (2015) Self-healing materials: A review of advances in materials, evaluation, characterization and monitoring techniques. Compos Part B: Eng 87. https://doi.org/10.1016/j.compositesb.2015.09.057
117. Qureshi S, Anjum S, Hussain M, Sheikh A, Gupta G, Almoyad MAA, Wahab S, Kesharwani P (2024) A recent insight of applications of gold nanoparticles in glioblastoma multiforme therapy. Int J Pharm 660:124301. https://doi.org/10.1016/j.ijpharm.2024.124301
118. Hao Y, Zhang B, Zheng C, Ji R, Ren X, Guo F, Sun S, Shi J, Zhang H, Zhang Z, Wang L, Zhang Y (2015) The tumor-targeting core-shell structured DTX-loaded plga@au nanoparticles for chemo-photothermal therapy and X-ray imaging. J Control Release 220(Pt A):545–555. https://doi.org/10.1016/j.jconrel.2015.11.016
119. Yasri S, Wiwanitkit V (2017) Important ethical issues for nanomedicine. J Res Med Sciences: Official J Isfahan Univ Med Sci 22:138. https://doi.org/10.4103/jrms.JRMS_856_17
120. Singh N, Manshian B, Jenkins GJ, Griffiths SM, Williams PM, Maffeis TG, Wright CJ, Doak SH (2009) Nanogenotoxicology: the DNA damaging potential of engineered nanomaterials. Biomaterials 30(23–24):3891–3914. https://doi.org/10.1016/j.biomaterials.2009.04.009
121. Erickson A, Jackson LR, Camphausen K, Krauze AV (2024) Mucins as precision biomarkers in glioma: emerging evidence for their potential in biospecimen analysis and outcome prediction. Biomedicines 12(12):2806. https://doi.org/10.3390/biomedicines12122806
122. Banerjee D, Harfouche R, Sengupta S (2011) Nanotechnology-mediated targeting of tumor angiogenesis. Vasc Cell 3(1):3. https://doi.org/10.1186/2045-824X-3-3

Publisher's note Springer Nature remains neutral with regard to jurisdictional claims in published maps and institutional affiliations.

Springer Nature or its licensor (e.g. a society or other partner) holds exclusive rights to this article under a publishing agreement with the author(s) or other rightsholder(s); author self-archiving of the accepted manuscript version of this article is solely governed by the terms of such publishing agreement and applicable law.

UNCORRECTED PROOF

Authors and Affiliations

Giorgia De Rosa[1] · Marco Zeppieri[2,3] · Caterina Gagliano[4,5] · Alessandro Tel[6] · Daniele Tognetto[3] · Pier Paolo Panciani[1] · Marco Maria Fontanella[1] · Tamara Ius[7] · Edoardo Agosti[1]

Tamara Ius
tamara.ius@gmail.com

Giorgia De Rosa
g.derosa003@studenti.unibs.it

Marco Zeppieri
markzeppieri@hotmail.com

Caterina Gagliano
caterina.gagliano@unikore.it

Alessandro Tel
alessandro.tel@icloud.com

Daniele Tognetto
tognetto@units.it

Pier Paolo Panciani
pierpaolo.panciani@unibs.it

Marco Maria Fontanella
marco.fontanella@unibs.it

Edoardo Agosti
edoardo_agosti@libero.it

1 Division of Neurosurgery, Department of Medical and Surgical Specialties, Radiological Sciences and Public Health, University of Brescia, Piazza Spedali Civili 1, Brescia 25123, Italy

2 Department of Ophthalmology, University Hospital of Udine, p.le S. Maria della Misericordia 15, Udine 33100, Italy

3 Department of Medicine, Surgery and Health Sciences, University of Trieste, Trieste 34127, Italy

4 Department of Medicine and Surgery, University of Enna"Kore", Piazza dell'Università, Enna 94100, Italy

5 Mediterranean Foundation"G.B. Morgagni", Catania 95125, Italy

6 Clinic of Maxillofacial Surgery, Head-Neck and NeuroScience Department, University Hospital of Udine, Udine, Italy

7 Academic Neurosurgery, Department of Neurosciences, University of Padova, Padova 35121, Italy

UNCORRECTED PROOF

Journal: **10072**
Article: **8457**

AQ1. Please check if author names and affiliations are correctly presented; otherwise, please amend.

AQ2. Kindly check and confirm whether the corresponding authors and thier affiliation are correctly identified.

AQ3. Please check if section headings are assigned to correct levels; otherwise, kindly amend.

AQ4. References [115], [116], [117], [118], [119], [120], [121] and [122] were provided in the reference list; however, these were not mentioned or cited in the manuscript. As a rule, if a citation is present in the text, then it should be present in the list. Please provide the location of where to insert the reference citation in the main body text. Kindly ensure that all references are cited in ascending numerical order.
